# Causality assessment of adverse events following immunization: the problem of multifactorial pathology

**DOI:** 10.12688/f1000research.22600.2

**Published:** 2020-04-14

**Authors:** Paolo Bellavite

**Affiliations:** 1 Department of Medicine, Section of General Pathology, University of Verona Medical School, Verona, 37134, Italy

**Keywords:** Vaccination, Adverse events following immunization, Inflammation, Autoimmunity, Genetic susceptibility, Multifactorial diseases, Mandatory vaccinations, Injury compensation

## Abstract

The analysis of Adverse Events Following Immunization (AEFI) is important in a balanced epidemiological evaluation of vaccines and in the issues related to vaccine injury compensation programs. The majority of adverse reactions to vaccines occur as excessive or biased inflammatory and immune responses. These unwanted phenomena, occasionally severe, are associated with many different endogenous and exogenous factors, which often interact in complex ways. The confirmation or denial of the causal link between an AEFI and vaccination is determined pursuant to WHO guidelines, which propose a four-step analysis and algorithmic diagramming. The evaluation process from the onset considers all possible “other causes” that might explain the AEFI and thus exclude the role of the vaccine. Subsequently, even if there was biological plausibility and temporal compatibility for a causal association between the vaccine and the AEFI, the guidelines ask to look for any possible evidence that the vaccine could not have caused that event. Such an algorithmic method presents several concerns that are discussed here, in the light of the multifactorial nature of the inflammatory and immune pathologies induced by vaccines, including emerging knowledge of genetic susceptibility to adverse effects. It is proposed that the causality assessment could exclude a consistent association of the adverse event with the vaccine only when the presumed "other cause" is independent of an interaction with the vaccine. Furthermore, the scientific literature should be viewed not as an exclusion criterion but as a comprehensive analysis of all the evidence for or against the role of the vaccine in causing an adverse reaction. Given these inadequacies in the evaluation of multifactorial diseases, the WHO guidelines need to be reevaluated and revised. These issues are discussed in relation to the laws that, in some countries, regulate the mandatory vaccinations and the compensation for those who have suffered serious adverse effects.

## Introduction

Public health policy supports broad vaccination and, at the same time, acknowledges the prospect of adverse events following immunization (AEFI), harming a few individuals. Most countries have introduced laws that allow compensation for people who think they have been seriously and/or permanently damaged by recommended or mandatory vaccines, or for families in case of death. An AEFI is defined as “ *any untoward medical occurrence which follows immunization and which does not necessarily have a causal relationship with the administration of the vaccine. The adverse event may be any unfavorable or unintended indication, abnormal laboratory finding, a symptom or a disease.*” ^1^. Although the rules introduced by different countries often diverge ^2– 4^, an essential part of the evaluation of AEFI is the search for whether or not there is a causal link between the administered vaccine and the subsequent pathological phenomenon. It is evident that in this area, the causality assessment plays a crucial role for both public health policies and any possibly injured individuals.

WHO guidelines for causality assessment ^1^, provide that “ *Allegations that vaccines/vaccination causes adverse events must be dealt with rapidly and effectively. Failure to do so can undermine confidence in a vaccine and ultimately have dramatic consequences for immunization coverage and disease incidence long after proof is generated that the adverse event was not caused by a vaccine (e.g. autism and MMR, encephalopathy and pertussis)*.” This articulation is entirely understandable and plausible. The same guidelines do not mention the affected individual for whom a causal inquiry is important, as in the absence of damage causation recognition, the harmed individual cannot access any compensation by provided programs. In addition to generating an obvious injustice, too rigid and restrictive rules could undermine the confidence of the population in the vaccine solution, create an expectation of claim denial and lead paradoxically to a decline in coverage. Present trends in several countries towards an increasing number of mandatory vaccines, is a delicate and controversial subject, impacting both social and economic concerns ^5^. This is another reason why it is important that the procedure of causality assessment is accurate in theory and practice.

Causality is the relationship between two events (the cause and effect), wherein the second event is a consequence of the first. The WHO guidelines ^1^ acknowledge that “ *Sometimes there are multiple factors that may precipitate the effect (event) or may function as co-factors so the side effect (event) occurs.*” As far as vaccines are concerned, the fact that severe reactions affect only a few individuals suggests in most cases vaccines are not the only cause of the event and further factors are necessary in the development of pathology.

The growth of multifactorial diseases in the last decades has led to the development of the “medicine of complexity” ^6^, ranging from cardiology ^7^ to epidemiology ^8, 9^, from pharmacology ^10^ to nursing care ^11^, or forensic medicine ^12^. To underline the importance of the topic in modern medicine, in 2002 the journal *Science* dedicated a whole issue (vol. 296, n. 5568) to the “puzzle of complex diseases”, including papers on the causes of diabetes ^13^, systemic lupus erythematosus (SLE) ^14^, schizophrenia ^15^, and considering the challenges of sorting out the multiple genetic, infectious and life-style factors and their interaction in the pathogenesis of common diseases. The pathogenesis of autoimmune disease is characterized by a complex interaction between genetic and environmental factors, and immune and hormonal reactions, which is the much talked of “mosaic of autoimmunity” ^16^. In vaccinology, the new developing fields of “vaccinomics” and “adversomics” exploit the powerful tools of bioinformatics to study adverse side effects to vaccines, using a systems biology approach ^17– 22^. Furthermore, disorders characterized by episodes of exaggerated inflammatory response “hyperinflammatory states” or “autoinflammatory syndromes” develop as multifactorial diseases, affecting the severity and frequency of clinical findings ^23, 24^.

To ensure compliance with the above criteria and wider acceptance of the results, the WHO recommends that the assessment of AEFI causality is performed by a multidisciplinary committee comprised of experts from paediatrics, neurology, general medicine, forensic medicine, pathology, microbiology, immunology and epidemiology. In this opinion article, the problem is addressed from the standpoint of general pathology and immunopathology. In order to frame the correct perspective and scientifically founded causation assessment, it is appropriate to summarize the main mechanisms of vaccines and the possible reasons for a severe adverse reaction. This knowledge is essential in order to properly utilize the WHO algorithm, where the plausibility and temporal compatibility of an AEFI is evaluated.

## The complexity of reactions to vaccines

Vaccines are mixtures of substances that cause milder forms of diseases, or mimic those of real diseases and, therefore can cause harm. The latest publication of the Italian Drug Agency (AIFA) July 30, 2019 ( https://www.aifa.gov.it/), reports that serious AEFI correlated to vaccines in 2018 were 3.1 per 100,000 doses, with considerable differences between the different vaccines, for example the vaccine MPRV correlated with a rate of 12.7 reports per 100,000 doses. On the other hand, there is evidence of significant differences in rates of AEFI, according to the methods of data collection. A recent paper reported a notification rate of 3,800 correlated adverse events per 100,000 doses of measles/mumps/rubella/varicella (MMRV) vaccine (often administered together with anti-hepatitis A) ^25^. The latter publication states that the use of “active” reports data is essential for the study of adverse events defined as “rare” (those whose prevalence is less than 1/1,000 doses).

**Figure 1.**
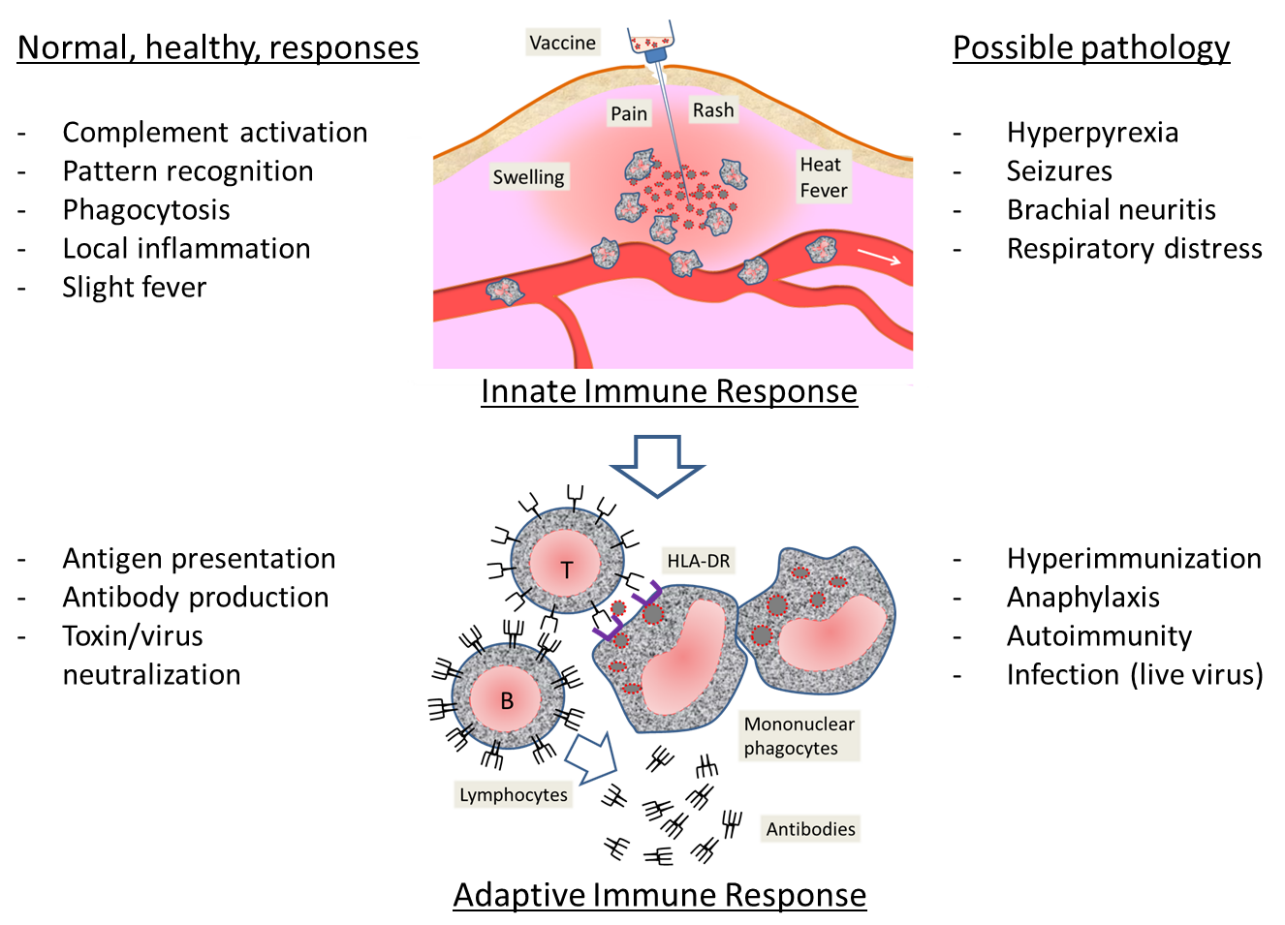
Innate and adaptive responses to a vaccine. Left column: normal responses; right column: possible pathology (excess/disorder of biological responses). Image is author’s own, produced for this review.

A vaccine may cause serious adverse reactions for three reasons: a) the material, that is, the content is “defective” or “contaminated”, due to preparation or storage inaccuracies; b) administration errors, such as accidental intravenous injection, the injection near a nerve plexus or the delivery of aluminium in the skin instead of into muscle; c) abnormal “reaction”, manifesting excessive biological stress caused by a foreign material or live attenuated virus. The most severe reactions are related to the vaccine action that involves two types, an “innate immune response”, linked to the early biological defences to injected matter, and a more antigen-specific response, linked to the adaptive immune defences ( Figure 1).

### Innate immune responses

The first phase immediately following the inoculation of the foreign material is the activation of a local inflammatory reaction at the point of injection, primarily involving the phagocytic cells such as monocytes and macrophages; the inflammation certainly leads to the production of cytokines by epithelial, mesenchymal and nerve cells: the release of classical inflammatory cytokines such as IL-1, IL-6, TNF-alpha, occurs a few hours after injection of aluminium-adjuvanted vaccines ^26^. When the reaction is sufficiently strong, the local inflammatory mediators (complement, cytokines, chemotactic factors) spread and amplify the reaction at a systemic level, which explains the general and neurological symptoms in the first hours or days after the vaccine. There appears to be a strong relationship between increased concentration of cytokines and febrile reactions, lymphadenopathy and generalized rash, after yellow fever vaccination ^27– 29^.

In very general terms, the inflammatory phase becomes pathological when it is in “excess”, i.e. causes negative side effects that outweigh those necessary to achieve the protective and repairing purposes. As for vaccines, fever is a useful mechanism to promote the mobilization of cellular, vascular and metabolic defences, to kill viruses and activate immunity, but it becomes “disease” beyond a certain temperature (the so-called “hyperthermia”). Conventionally, hyperthermia superior at or in excess of 39.5°C is considered a severe adverse reaction to the vaccine, and may cause seizures. The risk of febrile seizure increases over 5 times in children aged 12 to 35 months, within 6 to 11 days after exposure to the measles/mumps/rubella (MMR) vaccine ^30, 31^. Compared with MMR alone, the MMRV vaccine doubles the risk of febrile seizures in children aged 10–24 months, and does not modify it in children between 4 and 6 years ^32^. Receipt of DTP vaccine, but not of DTaP ^33^, was associated with a 5-times increased risk of febrile seizures on the day of vaccination ^34^.

Febrile seizures represent a predominantly functional disorder and are generally considered relatively benign, i.e. do not leave organic brain damage. However, in the case of longer term high temperature and convulsions (complex seizures, defined as an episode >15 min or recurrence within 24 hours ^35^), or in patients with cardiovascular system diseases, the brain can suffer from disturbance even on the level of cell viability, related to inflammation itself (cell damage due to excitotoxicity or to oxygen metabolites secretion by microglia), respiratory distress and anoxia. In extreme cases, prolonged hyperpyretic seizure syndrome can result in brain degeneration and/or death ^36– 40^. As for the long-term consequences, the risk of developing epilepsy after complex febrile seizures is estimated at around 10–20% ^41^.

A particular role may involve simultaneous injections of vaccines, because it is obvious that the type of nonspecific reactions are enhanced, if they are due to additional pathogenic factors ^26^. This is even more evident when you consider that vaccination is generally not recommended in children who have a febrile condition, whatever the cause.

### Adaptive immune responses

The second step of the vaccine function is the activation of immune system through antigen presentation by mononuclear phagocytes to lymphocytes. At this stage, a pathologic reaction may consist of unwanted responses due to hyper-immunization, autoimmunity, allergy and damaging infection (the latter in case of live viruses in immunocompromised patients).

Hyper-immunization reactions for repetitions of the tetanus vaccine have been documented in the Italian population, where the prevalence of an excess of antibodies (>5 IU / ml) was described in 17% of the observed subjects ^42^. The diseases induced by hyper-immunization following the administration of vaccines are due to sensitivity to one of the components of the vaccine, and exacerbation of atopic or vasculitis symptoms ^43, 44^. If the status of pre-existing immunity is unknown, to avoid hyper-immunization and its risks, it is recommended to carry out laboratory testing to determine antibody titre and avoid vaccination if the titre is already high enough ^45^.

Vaccines have long been suspected of playing a negative role in inducing autoimmune diseases ^46– 56^. The most established connections between autoimmune disease and vaccinations have been reviewed ^57^ and include: immune thrombocytopenia after MMR vaccination, Guillain–Barré syndrome after swine influenza vaccination, reactive arthritis after hepatitis B and rabies vaccinations, SLE and other autoimmune diseases, after hepatitis B and human papilloma virus vaccinations.

Evaluation of the association of AEFI with autoimmune diseases is challenging due the complex innate and adaptive immune responses to vaccine components (adjuvants, antigens, preservatives) that may contribute to reactogenic responses ^17^. The specific components of the vaccines (antigens) can trigger immunity against microbial antigens, but also a self-immunity in the case of there being a molecular mimicry (similarity) between antigen protein sequences and protein sequences of components of the organism or HLA receptors ^16, 52, 58– 60^. The emergence of the post-vaccination autoimmune syndrome is associated with genetic predisposition, for example, HLA-DRB1 or HLA-DRB4, as a result of exposure to additional external factors or endogenous autoimmunity triggers ^61– 63^.

**Figure 2.**
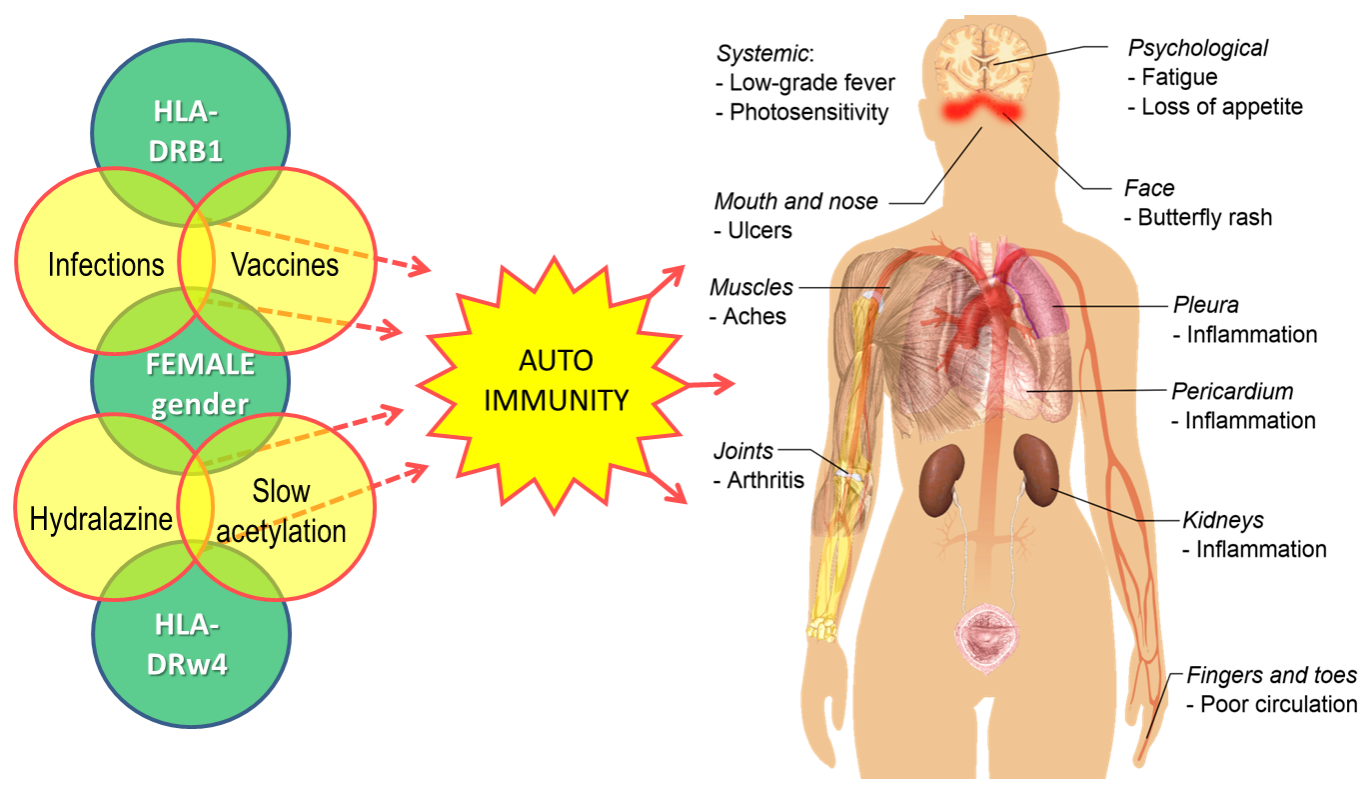
The multiple factors that may trigger autoimmunity in the pathogenesis of lupus erythematosus and the common signs and symptoms of the disease. The image of the body is by Mikael Häggström, used with permission ( in the public domain).

A typical autoimmune disease, which seems to be related to immunization in about a third of cases ^64, 65^, is SLE ( Figure 2). SLE pathogenesis is very complex, subject to both environmental factors – like viruses ^66^, bacteria ^67^, but also to drugs ^68^ (and this is highly significant) – and also to genetic susceptibility (for example HLA polymorphism) ^69, 70^ as well as hormonal factors (in fact, it has a considerable prevalence in the female gender) ^71^. Moreover, the vaccine adjuvants can increase the immunogenicity of the injected antigens and, as a consequence, may also increase the risk of triggering autoimmune adverse events ^56^.

According to some authors, it may be possible to conceive the production of vaccines based solely on unique sequences of pathogens, which could then frustrate the potential risk of cross-reactivity in the existing vaccine formulations ^72, 73^. Unfortunately, this approach is still utopian for vaccines currently in use.

Both local inflammatory responses and immune systemic responses are increased by aluminium adjuvants. Concerns about the safety of aluminium emerged as a result of the recognition of its biological persistence, exhibiting an unexpectedly long duration within immune and nervous system cells ^74– 82^. Aluminium adjuvant particles remain in the lymphoid organs and can even get into the brain, a phenomenon documented in animal models ^83, 84^. In particular, the long-term persistence of an aluminium granuloma, also defined as macrophagic myofasciitis, is characterized by chronic arthromyalgia, fatigue and cognitive dysfunction ^74, 85^. The problem is not just a difficulty in curing local granulomatous inflammation ^86^, but the systemic effects, such as oxidative stress on the blood ^87^, cognitive dysfunctions ^88, 89^, chronic fatigue syndrome ^90, 91^, hypotonia ^92^, child motor retardation ^93^, sensory disturbances, loss of vision and cerebellar signs ^94^, as well as alterations of cerebral circulation ^95^. A recent review illustrates several mechanisms by which aluminium introduced through immunizations can produce chronic neuropathology in genetically susceptible individuals ^96^. These authors recommend that the use of aluminium salts in vaccines be discontinued in favour of adjuvants less involved in the activation of autoinflammatory and neurolesive reactions.

### Genetic susceptibility

The mere fact that vaccines are followed by serious adverse effects in only a few individuals suggests that in most of these cases underlying susceptibility factors are present, which predispose or prepare the complex innate or adaptive reaction system for an excessive or biased reaction. Among these factors, it is obvious that genetically defined pre-existing conditions are at work in some groups of subjects in the population ^18^, but research in this field is in its infancy. A genetic predisposition results from specific genetic variations that are often inherited from a parent. These genetic changes contribute to the development of a disease but do not directly cause it.

The genetic conditions that have been associated with excessive vaccine reactions can be various ( Table 1), as congenital immunodeficiency, variants to the virus receptors and cytokines, epileptic tendencies, defects of detoxification inhibitors and enzymes, and so on. However, the small size of the groups, the often anecdotal type of the reports, and the multifactorial nature of diseases, do not allow us to draw definitive conclusions on causality regarding the observed associations between genetic types, vaccine and reported outcomes.

**Table 1.** Genetic disorders or variants that have been associated with adverse effects following immunization.

Condition	Vaccine	Possible disease	References
Primary immunodeficiency (AGG, CID, CVID, HGG, SCID)	OPV	Vaccine-derived polio	115
Primary immunodeficiency (SCID)	Rotavirus	Severe persistent diarrhoea, vomiting, failure to thrive	116– 118
Primary immunodeficiency (SCID)	BCG	Severe tuberculosis, death	119, 120
Primary immunodeficiency (CD8 deficit, dysgammaglobulinaemia)	MMR	Encephalitis	121
Polymorphisms of MBL and TLR receptors of innate immunity	BCG	Osteitis	122, 123
Polymorphism of purine receptor P2X7.	BCG	BCG lymphadenitis	124
Polymorphism of IL17A	BCG	Osteitis	125
Specific haplotypes in the MTHFR and IFR1	Smallpox	Generalized skin eruptions	126
Specific haplotypes in the IL1 and IL18 genes	Smallpox	Systemic symptoms, fever	127, 128
Polymorphism of IL-4	Smallpox	Decreases susceptibility to systemic adverse events	127, 129
SCN1A mutations	DTP	Epileptic encephalopathy	103, 130, 131
SCN1A, SCN1B or PCDH19 mutations (Dravet syndrome)	DTP, DtaP, and MMR	Epileptic seizures, autism-like symptoms	104, 132, 133
Polymorphisms of interferon-stimulated gene IFI44L and CD46 (receptor for measles virus)	MMR	Febrile seizures	101
SCN2A mutations	MMRV	Episodic ataxia, impaired speech development	134
Mutations in the catalytic subunit of PI3K	Varicella	Disseminated varicella	135
Mutation in IL17R	Varicella	Disseminated varicella	136
Polymorphisms in chemokine receptor CCR5 and its ligand RANTES genes	Yellow fever	Viscerotropic disease, multiple- organ system failure	137
HLA-DQB1*06:02 and polymorphism of T-cell receptor-alpha	AS03 adjuvanted A/H1N1	Narcolepsy	138, 139
Polymorphism of GDNF-AS1	AS03 adjuvanted A/H1N1	Narcolepsy	139
HLA-DRB1*01	Aluminium-hydroxide adjuvanted vaccines	Macrophagic myofasciitis	140
HLA-DRB1 (*01:01, *03:01, *04:01,*13:01, *15:01)	Hepatitis B	Autoimmunity	62, 141, 142
HLA-DRB1*1102/1132, DRB3*0202/0202, DQA1*0505/0505, DQB1*0301/0301	Hepatitis B	Systemic Lupus Erythematosus	70
Type 1 GSD	Any	Hypoglycaemia	143
Mitochondrial dysfunction, increased aspartate aminotransferase and serum creatine kinase	DTP; Haemophilus i. B; MMR; polio; varicella	Autism	144

AGG: agammaglobulinaemia; CID: combined immunodeficiency; CVID: common variable immunodeficiency; HGG: hypogammaglobulinaemia; SCID: severe combined immunodeficiency; MBL: mannose-binding lectin; TLR: toll-like receptor; MTHFR: 5,10-methylenetetrahydrofolate reductase; IRF1: interferon regulatory factor-1; SCN1A: sodium channel, voltage-gated, type I, alpha subunit; PCDH19: protocadherin 19; P2X7 is a purine (ATP) receptor; IL17R: interleukin-17 receptor; PI3K: phosphatidylinositol-3-kinase; AS03: adjuvant systems 03 (oil-in-water emulsion); GDNF-AS1: glial-derived neurotrophic factor antisense RNA-1; GSD: glycogen-storage disease.

Febrile seizures are genetically complex disorders, believed to be influenced by variations in several susceptibility genes ^97^ and, among the susceptibility genes, by those encoding cytokines of the acute phase ^98^. The risk of post-vaccination febrile seizures increased in subjects with previous and family history of febrile convulsions, showing that in some subjects there is a predisposition, which is obviously a co-factor in determining the risk of the vaccine ^99, 100^. The latter authors suggested that in order to reduce the risk of adverse reactions to MMRV, children with a family history of febrile seizures should not be vaccinated. Two loci were clearly associated with febrile seizures MMR-related, but not with those from other causes ^101^: the IFI44L interferon-stimulated gene and the CD46 receptor for measles virus. It is interesting that the same IFI44L and CD46 genes are among those that affect the magnitude of the antibody response to measles ^102^.

Among the genetic factors and in particular polymorphisms, the role of SCN1A gene mutations is demonstrated by the fact that the risk of post-vaccine seizures increased in patients with Dravet syndrome, a severe epileptic encephalopathy ^103– 105^. Vaccination is the trigger of the first seizure in about 50% of cases ^106^, while the vaccination program does not seem to increase long-term consequences of the disease on cognitive function. In children, genetic or structural defects are an underlying cause of epileptic seizures onset, after routine immunization, that may act as a triggering factor ^107^ and these authors suggested that early genetic testing should be considered in all children with vaccination-related onset of epilepsy. In the context of immunodeficiency diseases, it is important to point out that live vaccines may be safely administered to children with Di George syndrome, a congenital T-cell defect associated with a deletion in chromosome 22 (22q11. 2 deletion) ^108^.

As a first step towards a systematic collection of genetic susceptibility factors, Lin, He and Xie have created an ontological framework (Ontology of Genetic Susceptibility Factors, OGSF), which may provide guidance for representing diverse types of genetic susceptibility factors for vaccine adverse events, such as HLA alleles, SNPs, genes, and gene haplotypes ^109– 111^.

### The microbiome

In the complex mechanisms regulating the innate and adaptive responses to immunization, the role of the microbiome and of the intestinal barrier should be noted as important factors that may contribute to systemic inflammatory reactions ^112– 114^. Bacterial endotoxins that may be released from the intestine, in the case of increased intestinal permeability (also possibly caused by medication or dysmicrobism), should not be neglected. The interactions between products of the bacterial microbiome with immune cells trigger self-reactivity, chronic inflammation and tissue damage in genetically sensitive subjects ^145, 146^. The synergy between LPS and inflammatory cytokines is one of the simplest and most ubiquitous mechanisms of neuroinflammation and neurodegeneration ^114, 147– 149^. This can happen through the modification of substances by the intestinal bacterial flora, which can therefore become autoantigens and mistakenly trigger immune responses of the wall itself. In addition, recent studies have shown that a breakdown or increase in permeability of the intestinal barrier and the translocation of commensal bacteria or endotoxins into non-intestinal organs can trigger several autoimmune pathways ^112– 114, 150^. For example, many people with multiple sclerosis have been shown to have an altered microbiome, increased intestinal permeability and changes in bile acid metabolism ^151^. Allergic diseases and autoimmune encephalitis have also been correlated with changes in intestinal microbiota ^152^. For these reasons and as a precautionary measure, the healthy state of the gut should always be considered, before a vaccination procedure.

The role of microbiome is important from the perspective of susceptibility factors of AEFI, because it is possible that an alteration of the gut health, especially with the leak of endotoxins in the general circulation, increases the susceptibility to a stronger and more serious reaction to the immune stimulus represented by the vaccine . Under these predisposing conditions the plausibility that a serious inflammatory reaction may be triggered by a vaccination increases.

## WHO guidelines

**Figure 3.**
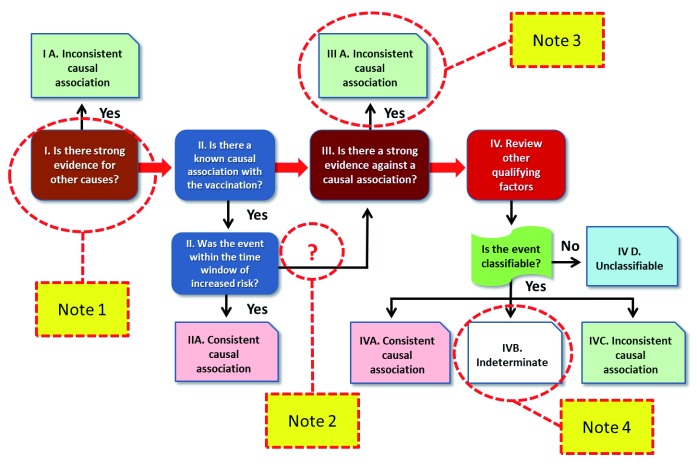
The WHO algorithm for causality assessment of AEFI, with the indicated notes discussed in this opinion article (yellow boxes).

WHO guidelines for causality assessment were published in 2013 ^153^ and updated in 2018 ^1^ ( https://www.who.int/vaccine_safety/publications/gvs_aefi/en/). The first step is to determine if the AEFI is “eligible”, meeting the minimum criteria for the assessment of causality, such as the presence of a clear diagnosis. The second phase (“checklist”) encompasses a systematic review of relevant and available information to deal with possible causal aspects of the AEFI. Then, an “algorithm” synthesizes the entire conceptual and methodological process in four steps ( Figure 3). Given the central importance of the WHO algorithm, for brevity and clarity of need, this article will focus on these steps, with four notes highlighting the main problems that emerge in the light of the previous discussion on the possible mechanisms of vaccination adverse reactions.

### Note 1. The “other causes”

The WHO algorithm of step 1 rules out the association of an AEFI with vaccination if there is another cause. This is the first and decisive criterion for exclusion and is stated on the “checklist”, alongside the question “ *Is there strong evidence for other causes?*” The text then further explains that a detailed medical history, clinical examinations and investigations, including laboratory tests on the patient, can help identify other conditions such as other diseases and congenital anomalies that may have caused the event. Provided is the example of the death of a girl, following vaccination against human papilloma virus (HPV), where a post-mortem examination accredited the cause to a malignant mediastinal tumour.

It should be noted that, in this first phase, the process systematically seeks another “strong” cause which, if found, would exclude the causal link. This concept of “strength” is not defined and can be misunderstood. According to medical historian Cosmacini ^154^, causality criteria are changing due to epidemiology of modern diseases: a *strong* causality criterion arises when the pathogenic cause, for example, the infectious agent, is “forcibly” followed by pathological effect, i.e. the disease or event; while a *weak* causality is when the cause has “ *less strength*” or “ *relative weakness*”. In these second possibilities, the relative weakness lies in the fact that the pathogenic cause or causes are probabilistically followed by the morbid effects. As explained above, in the presence of many possible causes, none of which is necessary or sufficient to determine that disease, the traditional concept of cause is transformed into the new concept of “ *risk factor*.” The multifactorial perspective, deriving from the observation of adverse reactions from the vaccine and from the recent knowledge of pathology and immunopathology (see above sections), means that the concept of “ *strength*” of causal association takes on a probabilistic meaning. Therefore, in the case of diseases involving several possible causes and/or mechanisms, the purpose of AEFI classification cannot be to identify "the" determining cause, but it may be more correct to try to establish with what probability one or more the factors involved (vaccines, genetic or epigenetic traits, previous or concomitant infections, drugs, age, gender, nutrition and metabolism, etc.) may have contributed to the occurrence of the event.

This is a very important aspect and should not be overlooked. If a disease is multifactorial in its nature, to be considered strong enough to exclude the contribution of a vaccine, the “other cause” must be *independent* of a possible synergistic interaction with the effects of the vaccine itself on the immune responses. For example, a complication of an advanced tumour, after pneumococcal vaccination can be considered “the” cause of death (as in the example provided by WHO guidelines), but a HLA haplotype that predisposes to autoimmunity cannot be considered “the” cause of an autoimmune disease that arose after the hepatitis vaccination.

### Note 2. Biological plausibility and the time window

The second step of the algorithm ( Figure 3) includes the evaluation of the “positive” elements that could be in favour of a consistent causal relationship, considering the biological plausibility and whether the event occurred inside a time window compatible with the risk after vaccination. If all these aspects are in favour of a role of the vaccine in the AEFI, without negative evidence, the causal association is classified as “consistent”. The biological plausibility of damage from the vaccine can be inferred by its known action: in some rare and susceptible individuals, an abnormal response to the stimulus, provided by antigens and adjuvants, unfortunately occurs. The person who has suffered damage from the vaccine usually was predisposed via some “risk” factor – genetic or acquired - which was not sufficiently “strong” to cause illness or injury. With vaccination moreover, the organism is destabilized leading to a pathological reaction. From this point of view, a steadier plausibility of a consistent association between disease and vaccine is generated, if two conditions occur: a) the same pathology (or a biologically similar one) has already been described in other cases after vaccination and b) the subject is a carrier of an increased proneness to that particular pathology.

Concerning the “time window” in which an AEFI can be considered as putatively associated with vaccination (box II in Figure 3), the questions are usually clear enough and should not pose significant issues, at least when previously described cases exist and for the “acute” cases (hours or days after vaccination). However, problems can be substantial when considering chronic illness and autoimmune disease, which can develop quite some time after vaccination. For autoimmune diseases, the difficulty is even greater because, as we have seen, they are “weakly” associated with vaccination. For example, a systematic review and meta-analysis suggests that vaccinations significantly increase the risk of SLE (RR=1.50; 95%CI 1. 05-2. 12, P=0. 02) and rheumatoid arthritis (RR=1. 32; 95%CI 1. 09-1. 60, P=0. 004) ^65^. This means, from a theoretical point of view, that about one in three SLE cases may also occur via the vaccine. However, among all people with SLE, we cannot say for whom the vaccine has had a causal role; we can only say, for a person who has had the appearance of SLE after vaccination, that there is about a 3 in 10 chance that vaccination has contributed negatively to the development of the disease.

It should be noted that eight patients with disseminated acute encephalomyelitis occurred less than 10 weeks after vaccination against hepatitis B (HBV) have been reported ^155^. Another case, which occurred 3 weeks after HBV vaccination, has been described more recently ^156^ and 93 patients with autoimmune disease after HBV vaccination have been reported ^47^. The mean latency period since the last dose of vaccine and the onset of symptoms was 43 days. However, studies of cohorts of children with neurological and behavioural syndromes (e.g. anorexia nervosa, attention deficit hyperactivity disorder, obsessive-compulsive disorder) have observed an increased risk of up to 12 months after vaccination ^157^. On the other hand, given the multifactorial nature of most chronic diseases, it cannot be excluded that, over a longer period, other causes determining the chronic pathology, unrelated to the vaccine, may well have occurred. Consequently, in the perspective of a single case, it is very probable that the undesired reactions which arose at a considerable distance from the vaccination escape the possibility of proving a causal association.

The WHO guidelines consider this problem under the question " *In this patient, did the event occur within a plausible time window after vaccine administration?*" and in a note cite as a " *detailed document*" a book of the Institute of Medicine ^158^. However, in that document there are no indications on the suitable time windows of autoimmune diseases or in general of chronic diseases following vaccinations. In some cases, causality is excluded by using rather short time windows. For example, the case of a man with symptoms of chronic inflammatory disseminated polyneuropathy that occurred 8 weeks after a tetanus toxoid vaccine ^159^ is presented and it is argued that this interval is "too long". However, autoimmune diseases and chronic post-vaccination syndromes in general can occur several weeks or months after vaccination. In cases of fibromyalgia and chronic fatigue disease following hepatitis B vaccination ^160^, the time interval between vaccination and the onset of symptoms was 38.6 days, but with a large time interval (+/- 79.4 days). In a systematic prospective case-referent study conducted to assess the risks of autoimmunity associated with HPV vaccines, a reasonable time window of 24 months for multiple sclerosis, connective tissue disease, type-1 diabetes, and thyroiditis was adopted ^161^. This paper excluded an association between HPV vaccination and these disease, but an increased percentage of cases had personal or family (in first-degree relatives) history of autoimmunity (14.7% of cases versus 7.2 % of referent group, p <0.05), endorsing the importance of genetic susceptibility to vaccine adverse effects.

Given the complexity and multifactoriality of chronic autoimmune diseases and the lack of precise references on the time frame of appearance of these diseases after vaccination, the possibility could be considered that, in an upcoming edition of the guidelines, it is specified that the time window for autoimmune diseases should be sufficiently large (e.g. 24 months ^161^) to not exclude slow-onset cases, or that a restricted time frame should be applied only to AEFI with acute onset (e.g. hyperthermia, febrile seizures, anaphylaxis).

Incidentally, it should be noted that the WHO algorithm published in 2018 ( Figure 3) lacks the indication “No” over the arrow connecting phase II box “ *Was the event within the time window of the risk?*” with the phase III box “ *Is there a strong evidence against a causal association?*”. As a matter of fact, in the algorithm of causation assessment published in 2013 there did appear the inscription “No” on the connection arrow arrow ^153, 162^. This omission in the recent algorithmic form can create misunderstandings, because those who follow the algorithm literally would be prevented from concluding with IIA (“ *Consistent causal association*”) and would necessarily proceed to phase III, a passage where again an exclusion criterion is offered, mostly linked to literature (see below). Thus the path proposed by the arrow would defeat any chance of reaching the IIA conclusion, although other evidence is favourable to a positive causation. To avoid any such misunderstanding, it would be more correct and reasonable to restore the previous descriptor “No” to the aforementioned connection.

### Note 3. The literature

In phase III of the algorithm, users must answer these key-questions “ *Is there strong evidence against a causal association?*” and “ *Is there a body of published evidence (systematic reviews, GACVS reviews, Cochrane reviews, etc.) against a causal association between the vaccine and the event?*” In practice, if there is published evidence in literature that rejects a statistically significant association between a disease and previous vaccinations, this argument could be used to exclude in each particular case that vaccination may have caused the reported disease. The evidence at population scale is used at individual level.

This is a criterion used in a very “strong” way, even to the point of excluding a case for lack of evidence of literature, leading to conclusion IIIA (“Inconsistent causal association”), even if in that particular case there is plausibility for a consistent association and a compatible time frame. The topics reported as emblematic are autism and sudden infant death syndrome (SIDS), as it is argued that according to the literature they cannot be caused by the vaccine. Specifically, the guidelines ^1^ write that “ *no evidence exists of a causal association between MMR vaccine and autism or autistic disorders*” and that “ *the committee concluded that vaccines did not cause SIDS*.” Here we must pay attention to language and related concepts, because the “lack of evidence of association” may become easily, but mistakenly, “evidence of the lack of association”. It would not be correct to use this lack of knowledge as a “guillotine” criterion to exclude causation in individual patients. In fact, epidemiological studies cited in the document may exclude an association at the population level but do not have the power to exclude rare cases, especially if the surveillance is not 100% efficient.

In a Cochrane review of 2012 ^30^ the design and reporting of safety outcomes in MMR vaccine studies, both pre- and post-marketing, are defined as *“largely inadequate”.* The question is how one can exclude any liability of the vaccine in contributing to the development of serious neurologic adverse effects, at least in sporadic cases of children predisposed via other genetic factors. The genetic background of autism is known (about a quarter of cases of autism have a genetic basis although only in rare cases is the disease totally genetic) but encephalopathy may also be determined by autoimmunity ^163– 167^, which, in turn, depends on some factor of an antigenic nature. It should also be noted that “autism” is not a disease with specific symptoms and reproducible in all subjects with personality disorders, so that we speak of “autism spectrum disorders”. The most obvious case of a possible overlap between autism spectrum symptoms and another disease, surely caused by vaccine adjuvants, is the macrophagic myofasciitis ^168– 171^. This topic was developed in a previous chapter, where the pathogenic effects of aluminium on the central nervous system were described, including cognitive dysfunction, sensory disturbances, and motor retardation.

Regarding SIDS (otherwise known as SUDC, sudden unexplained death in childhood), the considerations are partly different. SIDS (or SUDC) is strictly defined as “ *unexplained crib death from known causes upon autopsy*” ^38^. The prevailing literature, as rightly reported by WHO, states there is no association between SIDS, SUDC and vaccination. This is completely obvious, because if in a case of “death in a cradle” there is no symptom of particular diseases, nor any autopsy findings that can highlight the cause, this means that the role of the vaccine cannot be proved either. On the other hand, what the WHO algorithm can’t exclude is a violent adverse reaction to the vaccine components that can result in death in particularly fragile subjects. This type of reaction would not correspond to the definition of “SIDS” or “SUDC”. For this reason, in the case of a sudden death of a child after vaccination, before adopting the exclusion criteria of Phase III, the analysis of the case should exclude any clinical evidence (e.g. high fever, convulsions, respiratory distress, syncope) and autopsy finding (e.g. cerebral congestion, pneumonia, isolation of vaccine virus strain, significant increase of some cytokines in the blood) of strong inflammatory reactions. It was reported that in six cases of children who died in the crib after hexavalent vaccine and were previously diagnosed as “SIDS”, the autopsy revealed severe signs of encephalitis and other laboratory data indicating systemic inflammation ^172^. The literature does not exclude the fact that pneumonia is rarely as a result of vaccination, since cases of the syndrome have been reported in deceased patients after DPT vaccination ^173^, influenza ^174^, and anti-haemophilus influenzae type b ^175^. In summary, the benchmark linked to the scientific literature is important for the final categorisation (step IV), but should not be considered as a decisive criterion leading to conclusion IIIa, i.e. to exclude causal association in individual cases.

The WHO manual of causality assessment refers to the peer reviewed literature to evaluate whether there is evidence of association between vaccine and pathology (step 2 of the algorithm, see Figure 3) or if there is opposing evidence (step 3). However, this utilization of supposed “evidence” may be flawed, since the safety of vaccines is normally proven with clinical trials that are not conducted by comparisons with a true placebo, such as physiological solution (0.9% NaCl). The latter is the “gold standard” placebo against which the safety of all vaccines should be tested and ensured, but the reality is different, especially for those vaccines that contain adjuvants. For example, the safety of the HPV vaccine Gardasil was tested in 6 clinical trials, in 5 of which the control group received Aluminium Hydroxyphosphate Sulfate, while in only one of which the physiological solution was used as placebo. However, in the summary of the safety profile of the vaccine (available from: http://www.merck.com/product/usa/pi_circulars/g/gardasil/gardasil_pi.pdf, accessed 2020 April 2), the systemic and serious adverse effects, namely the rate of autoimmune disorders, are evaluated comparing the group receiving Gardasil with only one group, receiving aluminium or placebo. By this way, any potential reactogenic effect of aluminium salts was masked. Certainly, vaccine safety is assessed also in the post-marketing phase by means of pharmacovigilance systems, which can provide important indications on the incidence of AEFI in vaccinated subjects, which can be compared with unvaccinated subjects. However, this type of comparison is largely subject to various types of selection bias and to the diversity of non-randomized groups. The problem becomes even more serious in the case of adverse reactions with low incidence. For these methodological reasons, the application of the evidence from medical literature to assess causality should be used with great caution and should not become a cut-off argument to establish or exclude causality.

### Note 4. The final categorisation

Eventually, the evaluation process ends with a global assessment, according to four categories: “consistent” when the causal association between the event and the vaccine is considered plausible, “inconsistent” in the presence of other causes which can justify the event, “indeterminate” when the evidence is insufficient to support a causal relationship in the presence of confounding factors, and “unclassifiable” when the information necessary to carry out the assessment is inadequate.

Apart for the “unclassifiable” cases, for which there is no possible classification, the other categories must be discussed and weighed carefully. The distinction between “consistent” and “inconsistent” could possibly apply to some clear-cut cases, but it becomes forced when you see the adverse reaction to the vaccine manifesting itself as a complex and multifactorial process, wherein the predisposing conditions and the trigger are contributory causes, with different pathogenic mechanisms. The perspective adopted in this report implies that although different causes contribute to an adverse event following immunization, they cannot be considered necessary and sufficient “causes” of the event *per se* except in very special cases. This problem is not just a difficulty of language and definition, but reveals the conceptual approach adopted by WHO in the preparation of the guidelines in question, under which one proceeds “by exclusion”, in search of an “other cause“. But if the abnormal reaction to the vaccine (which has already been made in the diagnosis) has a multifactorial origin, proceeding by elimination of one or more con-causes is incorrect from a scientific perspective. For example, if a child affected by a serious heart condition, dies the day after vaccination, which led to strong fever and/or difficulty breathing, the most plausible hypothesis is that the effect was determined by the “cooperation” of two factors, both important and interacting, but none of which alone could explain the event, without the other. This point was already raised by Puliyel, Naik and Phadke who noted that, according to the WHO algorithm, a cardiac decompensation in children with an underlying heart disease “ *would not be considered causally related to the vaccine, although vaccination contributed to cardiac failure*” ^162, 176^.

As mentioned, multifactorial diseases, such as autoimmune diseases, are often conditioned by various genetic and acquired factors. In these cases, the role of vaccination could be to slatentize a predisposing condition, which would have led to the disease slower or would not even appear. If this is the case, it is probable that the case study will neither confirm nor deny the role of the vaccine, so that the causality assessment would come to the conclusion of an "undetermined" association. Obviously, this procedure, if applied systematically to a series of cases, would lead to an underestimation of the etiological role of vaccines in autoimmune diseases. To overcome this vicious circle, in the final categorization (phase IV), the probability that the vaccine played a role in determining the event could be assessed and scored, taking into account the other possible factors involved. In this way, it would prevent information on the partial role of the vaccine, obtained from a particular case, from being lost in the study of a series of cases.

A problematic approach to the causal assessment appears where the guidelines ^1^ state that “ *In doing causality assessment on an individual case report, it must be remembered that in essence one is conducting a differential diagnosis*" (page 7) and that “ *it is important to recognize that causality assessment of an AEFI in an individual patient is an exercise in medical differential diagnosis. A good clinician does not diagnose diabetes or coronary artery disease on the basis of conflicting or vague information. In the same way, an AEFI should not be causally linked to a vaccine without adequate information*" (page 34). Giving examples of such “differential diagnosis” can be misleading, because normal clinical activity is very different from causality assessment. In fact, the “ *differential diagnosis*” of a multifactorial disease normally is not based on the cause but on its clinical manifestation, that is, the signs and symptoms, the pathological findings, and laboratory results. On the other hand, in the case of an AEFI one is not conducting a “diagnosis” of the disease (also here the WHO procedure provides for a diagnosis be made from the beginning, otherwise the case is not even “eligible”), but is trying to determine what was the sole cause or were the plural causes of the reported adverse event.

The WHO causality assessment is mainly based on a direct ‘a cause-and-effect relationship” without taking into account the multifactorial nature of inflammatory and immune phenomena. By undervaluing interacting causalities, the method classifies a causal association as “inconsistent” when there is another cause and as “indeterminate” when the vaccine may be a cause but there is no proof that the vaccine is “the” cause. Using this definition of causal association, many adverse events, where the vaccine plays a role as con-cause, remain unrecognized. Others ^162^ have noted that, according to this scheme, an acute cardiac decompensation after influenza vaccination in an elderly person with chronic cardiac failure might not be considered as causally related to the vaccine. Similarly, sudden death after vaccination of an infant with pre-existing heart disease might not have relationship with the vaccine. Furthermore, the contribution of vaccine in precipitating encephalopathy in patients who are susceptible on account of genetic factors will also not be considered. If this type of problem occurs, in addition to causing detriment to a injured person, it leads to an overall underestimation of the risks of a given vaccine.

To illustrate how the application of the WHO algorithm is difficult and potentially error-prone, three case studies are presented ( Box 1) in which the death of children occurred within a short period of time after vaccination. These cases are described in the AIFA reports of vaccine surveillance of AEFI relating to 2016, 2017 and 2018 years ( https://www.aifa.gov.it/rapporto-vaccini). In all reported cases the causality link with the vaccine was excluded because of the presence of “other causes”. These examples raise some questions and deserve clarification, without which a high risk of misinterpretation exists. The notes of the author concern a) whether the alternative “other cause” was sufficiently clear and “strong” as a diagnosis and as a possible cause of death and b) whether or not there could be a plausible interaction between the pre-existing clinical conditions and the biological action of the vaccine.

Box 1. Childhood mortality cases reported by AIFA *

**Case 1:**

Case cited in AIFA 2017 Report for 2016: “ *Preterm baby girl (born after 34 weeks growth in utero), vaccinated at 11 weeks with Infanrix hexa, Prevenar 13 and Rotarix. (. . .). The death occurred about 20 hours after vaccination, due to sudden death classified by the whistle-blower as “death in a cradle”. The autopsy study revealed signs of pulmonary and meningeal congestion and a finding of liver vacuolization compatible with lipid metabolism disease. The causal link was not correlated with vaccination, due to the detection of another possible known cause of death (congenital defect of lipid metabolism) [references 1– 3].*”

In this case, the role of another possible known cause of death seems to be clearly described: the autopsy finding of “liver vacuolization” as “compatible” with the lipid metabolism disorder, but it is certainly not an accurate diagnosis. Given that the autopsy showed “lung congestion and meningitis,” how does this finding connect with an inborn error of lipid metabolism, which until then had presented no clear symptoms and was never diagnosed? To justify the possible “death in crib”, the genetic variant apoEe4 ^177^, is then cited in bibliography no. 3 leaving it to be understood that this defect could be the cause of death in the cradle. However, given a careful reading of the work, apoEe4 has equal prevalence in children with SIDS and healthy children. Furthermore, apoEe4 does not appear to cause liver vacuolization. Finally, in the hypothesis that the girl had a disease of lipid metabolism, how is it excluded that 3 different vaccines injected simultaneously may have been the trigger, since signs of lung and meningeal congestion were detected (more compatible with systemic inflammation than with lipid metabolism disease)? It is known that respiratory distress is described as an adverse reaction to hexavalent and encephalitis (remember meningeal congestion) and in rare cases has been associated with vaccination ^131, 178^. It should be remembered, moreover, the vaccine data sheet hexavalent provides that “ *when Infanrix hexa is co-administered with a pneumococcal conjugate vaccine or with the vaccine MPRV, the rate of febrile reactions is higher in comparison to what occurs as a result the administration of Infanrix hexa alone* “ and that on the data sheet of the vaccine Rotarix, it is indicated that vaccination “ *should be postponed in babies, who have a sudden high fever, diarrhoea or vomiting*. “ In brief, the question is whether or not, in a case like this, the “other cause” is “strong” enough to exclude the possible pathogenic effects of the vaccine, when the autopsy and the time window are compatible.

**Case 2:**

Case cited in AIFA Reports for 2016 and 2017: “ *20-month-old infant vaccinated with Neisvac-C. Two days after the administration of Neisvac C, reported feverish rise followed by death after a few hours. The whistle-blower reports that, at the time of the sanitary intervention, it was only possible to ascertain death. The death was diagnosed as “Sudden Unexplained Death in Childhood, SUDC” as a result of “hyperpyretic hyporeactivity” in the course of respiratory infection with viral aetiology and body temperature at the time of death at 41°C, arisen 52/53 hours after vaccination. Considering this evaluation, the causal link is “not related” to vaccination due to the simultaneous presence of another cause*.”

The SUDC is by definition “ *the sudden and unexpected death of more than one year old postnatal child that remains unexplained after a review of the medical history, the circumstances of the death and a complete autopsy*” ^38^. So, how this “diagnosis” of SUDC is to be reconciled with “respiratory infection with viral aetiology,” remains to be clarified. Either it is respiratory infection, or a SUDC. Moreover, the attribution of the pathology to a viral aetiology remains unexplained, given that no virus was isolated and that “at the time of the intervention of the health workers, it was only possible to ascertain death”. Finally, the “ *hyperpyretic hyporeactivity*” as a possible cause of SUDC is totally speculative, given that this condition/diagnosis/symptom is not described in any scientific literature. In this case, there is biological plausibility and a time window compatible with a pathogenic role of the vaccine in the triggering of an extremely strong inflammatory systemic reaction: a strong fever is a very common consequence (1–7% of cases) of vaccination with Neisvac-C and which can occur in the first 6 days ^179^. Why was the causal link excluded *a priori* without considering the vaccine at least as a “contributory” cause?

**Case 3:**

Case reported succinctly in the report AIFA 2018: Serious adverse reaction to hexavalent plus pneumococcal vaccines, where death was found “ *not related on the basis of available information*”: *6-month-old male patient with Down syndrome and congenital heart disease diagnosed as Fallot tetralogy associated with a complete atrioventricular septal defect-Rastelli type A, already subject to hypoxic crises in relation to paraphysiological stimuli*”.

Down syndrome itself does not cause death; heart disease is certainly a potentially fatal condition but often has a chronic course so that it has also been treated surgically, even in a patient with Down syndrome ^180^. However, in this case there is no certainty about the cause of death, i.e. evidence that the congenital malformation resulted in death on that occasion, independently of the vaccine delivered right before. No autopsy evidence is reported from which it can be understood if it was a hypoxic crisis, or signs of systemic, pulmonary or if cerebral inflammation had been detected. To rule out the role of the vaccine, in a case like this it would be important to know if the child had developed a fever, also in light of the fact that two vaccines were administered simultaneously (see note on Case 1). The problem is significant because if there had been systemic inflammation (reported by fever or other serum laboratory findings), the biological plausibility of the interaction with the vaccine would exist. In a child who certainly has a strong underlying pathophysiological fragility (" *hypoxic crises in relation to paraphysiological stimuli*"), how can it be excluded that simultaneous injection of hexavalent and pneumococcal vaccines (both of which are known to cause adverse reactions with respiratory diseases and therefore hypoxic crisis) may have contributed to the cardiovascular arrest? In a case like this, the “other cause” of death seems “strong”, but it is not “independent” of a possible triggering effect of the double vaccine.

* https://www.aifa.gov.it/rapporto-vaccini. The translation from the original Italian text is by the Author.

## Concluding remarks

Although all licensed vaccines are generally safe for the majority of people, the vaccinated may still suffer adverse events in reaction to various vaccines, a few of which can be serious or even fatal ^111, 181^. Regarding public health, the proper identification and classification of AEFIs allows for the most accurate information possible about the true frequency of certain ailments in combination with vaccines, thereby minimizing vaccine risks, reassuring the population and informing national or global public health strategies. Regarding the damaged individual, a sound causality assessment supports the affected individual (or family) pursuing any compensation scheme(s), if a consistent association of his/her illness with vaccination is demonstrated. Italian law, n. 210/1992, provides for such compensation in the form of a monthly allowance from the State to any person who has been, due to mandatory (or recommended) vaccination, the subject of injury or illness from which is derived a permanent impairment of physical and psychological integrity. In Italy, 691 people have been recognized as permanently damaged by vaccinations, including 27 deaths ( http://www.condav.it/).

This article has described the complexity and variety of adverse reactions to vaccines, from the perspective of general pathology and immunopathology. The consideration of the action mechanisms of vaccines, which is connected to the plausibility that a response to stress can be excessive or distorted in some cases, suggests that some aspects of the WHO procedure of causality assessment are inadequate to deal with this complexity. The rigid exclusion criteria which occurs in some steps of the algorithm (such as the “another cause” of AEFI in step I and the negative evidence in literature) can be a source of errors, or at least questionable interpretations, especially when the clinical situation or the autopsy are not clear and decisive. Error of evaluation would be to consider as “the cause” of AEFI any pathology that may be present at the time of vaccination, without considering the possible interaction between this pathology and the effect of the vaccine as a possible contributing cause.

Three case studies of causality assessment have been reported here ( Box 1), which led to an exclusion of the causal link. The few data that were officially communicated by the regulatory authorities (AIFA) in their reports do not allow direct criticism of the conclusions reached, but are sufficient to illustrate the difficulties and errors that may arise, in practical terms, in applying the algorithm in cases of complex clinical situations. Especially if the clinic or laboratory indicates that the AEFI derives from a multifactorial pathogenesis, it would not be correct to discard in this way a possible role of the vaccine in determining a serious adverse reaction, which obviously involves a series of conditions predisposing a body to damage. In fact, in the case of pre-existing or concomitant pathology, which can be considered a susceptibility factor, the vaccine could represent a contributory, triggering, or worsening condition.

From these considerations, a first operative suggestion emerges. To avoid potential errors of interpretation, it would be appropriate that in the WHO guidelines of the causality assessment be explicitly specified that the “ *other causes*” mentioned in step 1, should only be considered a reason for excluding the causal link, when they are “ *independent*” of the possible vaccine biological action. In other words, to declare “ *inconsistent*” the association with the vaccination, it should be *excluded* that the condition existing in the subject at the time of the damage may have *interacted* with the vaccine, enhancing its pathogenic potential (or vice versa the vaccine had worsened the pre-existing situation). Only in this situation, would it be correct to exclude the association between AEFI and vaccine action in phase I. In order for this prospect to materialize, the risk factors of vaccine reactions need to be better identified including additive (non-synergistic) and multiplicative (synergistic) forms, similarly to what has been done in other fields. Improved understanding of risk factors would contribute to reducing the uncertainties of vaccination choices, which are often perceived by the population as “leaps in the dark”.

A second topic of discussion concerns the claims for compensation for vaccination damage. Due to the inherent complexity of the pathogenesis of vaccination reactions, an absolute certainty of the causal role of the vaccine is always difficult, but often it is also difficult to exclude it. Using the WHO algorithm slavishly, it is likely that many adverse events, due to various concomitant factors, will end up as “ *indeterminate*”. This conclusion could gather many cases in which plausibly the vaccine damage may have occurred, but there is neither absolute certainty, nor adequate representation in the literature. For claimants, an “ *indeterminate*” causal link is equivalent in practice to the conclusion of “ *unrelated*” and is therefore potentially a reason for discrimination. The latter problem, with human and economic sides, could be addressed, for example, by considering the possibility of assigning compensation - perhaps in part - even if the hypothesis of vaccine damage is only “probable” and in any case its contributory role cannot be excluded.

The debate on the best methods of surveillance in the field of vaccinology should remain open, in the interest of the entire population. In the British Medical Journal, dr. Rebecca Chandler has asserted ^182^ that the target user group for the WHO classification system are persons working in countries in whom vaccines are administered via WHO sponsored public health programmes. Those persons are largely concerned with the detection of "signals" of changes in frequency of the more common, expected events which could suggest vaccine quality-related problems, immunisation errors, or multi-use vial contamination, etc. It seems from this that the WHO causality assessment is meant for poor and developing countries and most reports within the global database for pharmacovigilance have not been subject to WHO AEFI causality assessment. In contrast, higher income countries which do not rely upon implementation of vaccine administration through WHO public health programmes will handle reports of AEFI through these national pharmacovigilance centers. As a result, more general guidance is used for causality assessment, such as the Naranjo algorithm ^183, 184^ and the WHO-UMC criteria developed by various groups working within the greater field of pharmacovigilance. The WHO-UMC scale ( https://www.who.int/medicines/areas/quality_safety/safety_efficacy/WHOcausality_assessment.pdf) has been developed in consultation with the National Centres participating in the Programme for International Drug Monitoring and is meant as a practical tool for the assessment of case reports. It offers a simple methodology taking into account the clinical-pharmacological aspects of the case history and the quality of the documentation of the observation ^162^. Within this arrangement, other criteria such as previous knowledge and statistical chance play a less prominent role in the system, so the surveillance systems are better adapted for the detection of the rare and unexpected events.

Despite there is no universally accepted method for causality grading of adverse drug reactions, the WHO algorithm is now recommended specifically for the pharmacovigilance of vaccine adverse events and is increasingly used by researchers and epidemiologists worldwide, in Lower Middle Income Countries like India ^185, 186^ but even in developed countries. For example, the WHO causality assessment guidelines are widely utilized in Italy ^25, 187^, Germany ^188^, Canada ^189^, and were recommended by the Brighton Collaboration Group for analysis of safety data of vaccines in pregnancy ^190^. Given the importance and universal utilization of this approach and its inadequacies in the evaluation of multifactorial diseases, the WHO manual needs to be urgently reevaluated and revised.

In Italy, the WHO classification is considered the standard in the evaluation of AEFI originating from pharmacovigilance reports (see https://www.aifa.gov.it/sites/default/files/2018-04-09_Patrizia-Felicetti_sorveglianza_reazioni_avverse_vaccino.pdf), so it is normally the only one that is used for causality assessment. Of course, if people who believe they have suffered unrecognized damage from vaccination appeal to a court of justice, a much more detailed assessment follows, where the expert consultants of the parties are challenged with all the available documentation.

The difficulties that courts encounter when deciding on compensation claims in which scientific uncertainty is present are noteworthy, also because the case law of different countries like Germany and France diverge with regard to their relationship to scientific criteria of causality ^4^. In the Italian system, in the matter of civil liability, it is sufficient that the causal link between fact and harmful event occurred with a probability of 50% + 1 so that Civil Liability can be affirmed (See ex multis. Cass. Civ. Sent. N. 21619/2007). The aforementioned criteria have also applied to damages, deriving from compulsory (or strongly recommended) vaccinations. Indeed, the jurisprudence of the Supreme Court of Cassation has ratified the principle with multiple judgments (see Ex multis Cass. Civ. Sec. VI Judgment no. 25119/2017; Cass. Civ. Sez. Lavoro, Judgment no. 22078/2018) according to which the existence of the causal link between the vaccination administration and the occurrence of the damage to health must be evaluated according to a criterion of reasonable scientific probability inspired by the “more likely than not” principle. It is worth noting that in 2017 a judgement of the European Court of Justice in Luxembourg allowed courts to decide that a vaccine had caused harm, taking into account the “serious, specific, and consistent” presumptions of a causal relationship, even when there is no certain proof based on medical research to support this ^191^. The presumptions include the time frame between vaccination and the evidence of disease, a lack of family history of the disease, and a considerable number of instances of the disease appearing, after administration of the vaccine.

A third series of considerations concerns legislation in which a vaccination obligation is being imposed. Clearly, this imposition implies a small but not absent risk of adverse events. In certain high-risk groups, such as immunocompromised patients and those with a history of previous anaphylactic reaction to a vaccine or its components, selective withholding of immunizations must be considered to decrease potential adverse events. However, aside from the case of primary immunodeficiency and some rare metabolic disease, till now there has been no routine laboratory test available with sufficient predictivity power to detect an increased risk of adverse reactions at the individual level. In this situation, a cautionary criterion should be adopted for all cases in which the existence of susceptibility factors is suspected, such as for example: a) family pathobiographic history, i.e. the previous occurrence of serious adverse reactions in family members, even without managing to make a molecular diagnosis, or b) genetic variation in a precise sense, determined by some already known specific polymorphism (see Table 1). It seems reasonable that, if a child presents with an increased risk of adverse reactions compared to the average risk of the population, for this subject the vaccination obligation should be “loose” and the choice of whether to vaccinate (taking the risk of AEFI) or not to vaccinate (taking the risk of any illness to which the subjects are unprotected) should be left to the doctor, in agreement with the parents. A corollary of this problem indicates that in order to improve risk assessment, studies on genetic predispositions to vaccine damage should be increased by establishing systematic analysis programs for polymorphisms, to be carried out from birth. The more subjects that are entered in these databases, after years of accumulating cases and comparing healthy and damaged subjects, the more precise the calculation of the relative risk associated with vaccinations will be. As knowledge of vaccinomics and adversomics increases, this estimate will be an increasingly precise element in decision-making in the coming years.

Adverse reactions associated with the vaccine and immunization-related error events can affect healthy individuals and should be promptly identified to decide whether and how to compensate those affected. We must reiterate the need not to confuse the epidemiological and individual perspectives: one being the risk/benefit of vaccination, another is the recognition (and possible compensation) of a causal-link association between an adverse event and the vaccine. The fact that a child carries a heightened risk of vaccination, does not mean that he/she should not be vaccinated. The risk/benefit ratio must be weighed on an individual level with care and precision, especially considering the incidence and severity of diseases to which they would be exposed if unvaccinated. On the other hand, it is correct that damaged individuals - perhaps even those who just “probably” have suffered serious harm from vaccination – are recognized and compensated. This way of proceeding should also serve to increase the general confidence of the population in vaccinations and reduce litigation in the health care system.

## Data availability

No data are associated with this article.
