# Supplementary material for: Causality assessment of adverse events following immunization: the problem of multifactorial pathology
Source: F1000Res. 2020 Apr 14;9:170. Originally published 2020 Mar 9. [Version 2] doi: 10.12688/f1000research.22600.2 (PMC7111503; doi:10.12688/f1000research.22600.2)
Supplement: Peer review history

# Peer review: Reviewer response for version 2

**DOI:** 10.5256/f1000research.25635.r62234

**Published:** 2020-04-22

**Authors:** Jacob Puliyel

**Affiliations:** 1 Holy Family Hospital, New Delhi, Delhi, India

I think that section headings must describe the section contents and not be a reference, pointing to some portion of a Figure in the article.

 For example: ‘ **Note 1. The “other causes”**. This may be changed to **The “other causes”. (See Note 1). **This is only a suggestion. The manuscript is acceptable even if this change is not incorporated.

Is the topic of the opinion article discussed accurately in the context of the current literature?

Partly

Are arguments sufficiently supported by evidence from the published literature?

Partly

Are all factual statements correct and adequately supported by citations?

Partly

Are the conclusions drawn balanced and justified on the basis of the presented arguments?

Partly

Reviewer Expertise:

NA

I confirm that I have read this submission and believe that I have an appropriate level of expertise to confirm that it is of an acceptable scientific standard.

# Peer review: Reviewer response for version 2

**DOI:** 10.5256/f1000research.25635.r62232

**Published:** 2020-04-14

**Authors:** Christopher Exley

**Affiliations:** 1 The Birchall Centre, Lennard-Jones Laboratories, Keele University, Keele, UK

I am happy with the amendments.

Is the topic of the opinion article discussed accurately in the context of the current literature?

Partly

Are arguments sufficiently supported by evidence from the published literature?

Partly

Are all factual statements correct and adequately supported by citations?

Yes

Are the conclusions drawn balanced and justified on the basis of the presented arguments?

Partly

Reviewer Expertise:

I work on human exposure to aluminium and this includes an active research programme on the safety of aluminium adjuvants used in vaccinations.

I confirm that I have read this submission and believe that I have an appropriate level of expertise to confirm that it is of an acceptable scientific standard.

# Peer review: Reviewer response for version 1

**DOI:** 10.5256/f1000research.24947.r61076

**Published:** 2020-03-31

**Authors:** Rebecca Chandler

**Affiliations:** 1 Uppsala Monitoring Centre, Uppsala, Sweden

Causality assessment of adverse events following immunization: the problem of multifactorial pathology. 

 In his opinion piece the author has addressed the insufficiencies of the WHO AEFI causality algorithm to appreciate the complexities manner in which vaccines cause harm specifically those which are related to an “excessive or biased inflammatory and immune response” to the vaccine product. He writes from the perspective of a pathologist, specialists which are often involved in cases of compensation claims relating to individual level questions of causality. Of particular concern to the author, as demonstrated in the 3 case examples provided, are questions relating to the role of the vaccine as one of potentially multiple factors responsible for the clinical manifestation of an AEFI. In the words of the author: ”As far as vaccines are concerned, the fact that severe reactions affect only a few individuals suggests in most cases vaccines are not the only cause of the event and further factors are necessary in the development of pathology”. He makes a number of proposals for revisions to the causality algorithm: 1) causality assessment could exclude a consistent association of the adverse event with the vaccine only when the presumed "other cause" is independent of an interaction with the vaccine and 2) the scientific literature should be viewed not as an exclusion criterion but as a comprehensive analysis of all the evidence for or against the role of the vaccine in causing an adverse reaction.

 Overall, the opinion of the author is agreed. However, I believe that the manuscript would benefit from some acknowledgement or proposal for revision/clarification of the primary target users of the current WHO AEFI algorithm. The WHO AEFI causality assessment was developed by the Vaccines Safety Group at the WHO with the support of the Global Advisory Committee on Vaccine Safety. The target user group for this classification system are persons working in countries in whom vaccines are administered via WHO sponsored public health programmes (largely LMIC). Those persons are largely concerned with the detection of "signals" of changes in frequency of the more common, expected events which could suggest vaccine quality-related problems, immunisation errors, or multi-use vial contamination, etc. A prioritisation of the identification of such AEFI can be understandable to monitor the safety of vaccines for which there is several years of experience and safety surveillance from earlier use in HIC (such as conjugate pneumococcal vaccines, HPV vaccines). Within this framework, the burden of responsibility for surveillance for the rare and unexpected AEFI has fallen to countries (and in fact vaccine manufacturers) with greater resources.

 In contrast, higher income countries which do not rely upon implementation of vaccine administration through WHO public health programmes will handle reports of AEFI through these national pharmacovigilance centers. As a result, more general guidance is used for causality assessment, such as the WHO-UMC causality criteria and the Naranjo algorithm which developed by various groups working within the greater field of pharmacovigilance. Within such centers adverse event reports for drugs and vaccines are often maintained within a single database (one notable exception being the USA), and causality assessment is approached in a similar way for all products. Within this arrangement, the surveillance systems are better adapted for the detection of the rare and unexpected.

 I would like the author to reflect on the manner in which AEFI reports are handled in Italy, as it would be expected that vaccine reports originating from that country would be subject to collection, analysis and assessment standards endorsed by the European Medicines Agency. 

 With the increasing likelihood of the need to implement new vaccines directly into lower income settings, the requirement of surveillance systems in these countries to detect the “rare and unexpected” is apparent. The author could address this aspect as a way to emphasize the importance of the revisions proposed.

Is the topic of the opinion article discussed accurately in the context of the current literature?

Yes

Are arguments sufficiently supported by evidence from the published literature?

Yes

Are all factual statements correct and adequately supported by citations?

Yes

Are the conclusions drawn balanced and justified on the basis of the presented arguments?

Partly

Reviewer Expertise:

Pharmacovigilance, vaccine safety surveillance

I confirm that I have read this submission and believe that I have an appropriate level of expertise to confirm that it is of an acceptable scientific standard, however I have significant reservations, as outlined above.

# Response

**Published:** 2020-04-02

**Authors:** Paolo Bellavite

**Affiliations:** University of Verona School of Medicine, Italy

I am glad to see that overall, my opinion is agreed.  I thank for the suggestions of the Reviewer and I have revised my paper accordingly. I added two paragraphs to the text of Discussion:

 The debate on the best methods of surveillance in the field of vaccinology should remain open, in the interest of the entire population. In the British Medical Journal, dr. Rebecca Chandler has asserted Chandler, 2019 that the target user group for the WHO classification system are persons working in countries in whom vaccines are administered via WHO sponsored public health programmes. Those persons are largely concerned with the detection of "signals" of changes in frequency of the more common, expected events which could suggest vaccine quality-related problems, immunisation errors, or multi-use vial contamination, etc. It seems from this that the WHO causality assessment is meant for poor and developing countries and most reports within the global database for pharmacovigilance have not been subject to WHO AEFI causality assessment. In contrast, higher income countries which do not rely upon implementation of vaccine administration through WHO public health programmes will handle reports of AEFI through these national pharmacovigilance centers.  As a result, more general guidance is used for causality assessment, such as the Naranjo algorithm (Naranjo et al., 1981,Belhekar et al., 2014) and the WHO-UMC criteria developed by various groups working within the greater field of pharmacovigilance.  The WHO-UMC scale (https://www.who.int/medicines/areas/quality_safety/safety_efficacy/WHOcausality_assessment.pdf) has been developed in consultation with the National Centres participating in the Programme for International Drug Monitoring and is meant as a practical tool for the assessment of case reports. It offers a simple methodology taking into account the clinical-pharmacological aspects of the case history and the quality of the documentation of the observation (Puliyel and Naik, 2018).  Within this arrangement, other criteria such as previous knowledge and statistical chance play a less prominent role in the system, so the surveillance systems are better adapted for the detection of the rare and unexpected events.

 Despite there is no universally accepted method for causality grading of adverse drug reactions, the WHO algorithm is now recommended specifically for the pharmacovigilance of vaccine adverse events and is increasingly used by researchers and epidemiologists worldwide, in Lower Middle Income Countries like India (Singh et al., 2018; Sebastian et al., 2019) but even in developed countries. For example, the WHO causality assessment guidelines are widely utilized in Italy (Lombardi et al., 2019;Stefanizzi et al., 2019), Germany(Mentzer et al., 2018), Canada (MacDonald and Law, 2017), and were recommended by the Brighton Collaboration Group for analysis of safety data of vaccines in pregnancy (Jones et al., 2016). Given the importance and universal utilization of this approach and its inadequacies in the evaluation of multifactorial diseases, the WHO manual needs to be urgently reevaluated and revised.

 References

 Belhekar, M. N., S. R. Taur, and R. P. Munshi, 2014, A study of agreement between the Naranjo algorithm and WHO-UMC criteria for causality assessment of adverse drug reactions: Indian J Pharmacol, v. 46, no. 1, p. 117-120.

 Chandler, R. E., 2019, Modernising vaccine surveillance systems to improve detection of rare or poorly defined adverse events: BMJ, v. 365, p. l2268.

 Jones, C. E. et al., 2016, Guideline for collection, analysis and presentation of safety data in clinical trials of vaccines in pregnant women: Vaccine, v. 34, no. 49, p. 5998-6006.

 Lombardi, N. et al., 2019, Vaccines Safety in Children and in General Population: A Pharmacovigilance Study on Adverse Events Following Anti-Infective Vaccination in Italy: Front Pharmacol, v. 10.

 MacDonald, N. E., and B. J. Law, 2017, Le système de innocuité vaccinale canadien en huit étapes : des notions pour les travailleurs de la santé: Paediatr.Child Health, v. 22, no. 4, p. e17-e20.

 Mentzer, D., D. Oberle, and B. Keller-Stanislawski, 2018, Adverse events following immunisation with a meningococcal serogroup B vaccine: report from post-marketing surveillance, Germany, 2013 to 2016: Euro.Surveill, v. 23, no. 17.

 Naranjo, C. A., U. Busto, E. M. Sellers, P. Sandor, I. Ruiz, E. A. Roberts, E. Janecek, C. Domecq, and D. J. Greenblatt, 1981, A method for estimating the probability of adverse drug reactions: Clin.Pharmacol Ther., v. 30, no. 2, p. 239-245.

 Puliyel, J., and P. Naik, 2018, Revised World Health Organization (WHO)'s causality assessment of adverse events following immunization-a critique: F1000Res., v. 7, p. 243.

 Sebastian, J., P. Gurumurthy, M. D. Ravi, and M. Ramesh, 2019, Active surveillance of adverse events following immunization (AEFI): a prospective 3-year vaccine safety study: Ther.Adv.Vaccines Immunother., v. 7.

 Singh, A. K., A. L. Wagner, J. Joshi, B. F. Carlson, S. Aneja, and M. L. Boulton, 2018, Causality assessment of serious and severe adverse events following immunization in India: a 4-year practical experience: Expert.Rev.Vaccines, v. 17, no. 6, p. 555-562.

 Stefanizzi, P., P. Stella, D. Ancona, K. N. Malcangi, F. P. Bianchi, N. S. De, D. Ferorelli, C. A. Germinario, and S. Tafuri, 2019, Adverse Events Following Measles-Mumps-Rubella-Varicella Vaccination and the Case of Seizures: A Post Marketing Active Surveillance in Puglia Italian Region, 2017-2018: Vaccines (Basel), v. 7, no. 4.

# Peer review: Reviewer response for version 1

**DOI:** 10.5256/f1000research.24947.r61079

**Published:** 2020-03-23

**Authors:** Christopher Exley

**Affiliations:** 1 The Birchall Centre, Lennard-Jones Laboratories, Keele University, Keele, UK

There is one significant omission from this manuscript and it concerns the availability of quality peer reviewed science in the field of AEFI. 

 How do we know if a vaccine is the origin of AEFI if the vaccine has not been subject to clinical trials involving true placebos. The vehicle for most vaccines, probably all injected vaccines, is 0.9% NaCl. How many vaccine safety trials have used 0.9% NaCl as the placebo? The injection of 0.5mL of 0.9% NaCl IM or SC should be close to being 100% safe. The needle may damage a capillary and this could produce mild inflammation at the injection site. This could be uncomfortable for a short period of time, there may be some individuals where their genetic make-up means that the discomfort might last a little longer. However, for the vast majority of recipients, 99.9%, the injection of 0.5mL of 0.9% NaCl is absolutely safe. This is the gold standard placebo against which the safety of all vaccines should be tested and ensured. Of course, the reality is totally different. In my field of interest, vaccines that include an aluminium salt as an adjuvant, there have not been any clinical trials where a vaccine has been tested against a true placebo. I say this with the proviso that in one trial of the HPV vaccine Gardasil there is a report, filed with the Clinical Trials Database, where a small number of trial participants did receive a saline placebo. These individuals reported no (zero) AEFI, while the vaccine group and the Al adjuvant only group reported an incidence of ca 2.5% AEFI. 

 The reality is that the design of causality assessment of AEFI is purposely flawed to give both the impression that all vaccines are safe and, of course, to allow those conducting the trials (who are never independent) to decide which of the many and serious AEFI occurring regularly might be attributed to the vaccine. Vaccines are inherently unsafe and rather than investing in improving their safety the industry has devised a mechanism to disguise their toxicity. I do wonder why the author has not included some discussion of this in his otherwise well informed text.

Is the topic of the opinion article discussed accurately in the context of the current literature?

Partly

Are arguments sufficiently supported by evidence from the published literature?

Partly

Are all factual statements correct and adequately supported by citations?

Yes

Are the conclusions drawn balanced and justified on the basis of the presented arguments?

Partly

Reviewer Expertise:

I work on human exposure to aluminium and this includes an active research programme on the safety of aluminium adjuvants used in vaccinations.

I confirm that I have read this submission and believe that I have an appropriate level of expertise to state that I do not consider it to be of an acceptable scientific standard, for reasons outlined above.

# Response

**Published:** 2020-04-02

**Authors:** Paolo Bellavite

**Affiliations:** University of Verona School of Medicine, Italy

I acknowledge dr. Christopher Exley for his comment, asserting that there is one significant omission from the first edition of the paper, concerning the availability of quality peer reviewed science in the field of AEFI. I agree with his concerns, so I have included a specific paragraph at the end of the Note 3, devoted to the role of medical literature in the overall procedure :

 “The WHO manual  of causality assessment refers to the peer reviewed literature to evaluate whether there is evidence of association between vaccine and pathology (step 2 of the algorithm) or if there is opposing evidence (step 3). However, this utilization of supposed “evidence” may be flawed, since the safety of vaccines is normally proven with clinical trials that are not conducted by comparisons with a true placebo, such as physiological solution (0.9% NaCl). The latter is the “gold standard” placebo against which the safety of all vaccines should be tested and ensured, but the reality is different, especially for those vaccines that contain adjuvants. For example, the safety of the HPV vaccine Gardasil was tested in 6 clinical trials, in 5 of which the control group received Aluminum Hydroxyphosphate Sulfate, while in only one of which the physiological solution was used as placebo. However, in the summary of the safety profile of the vaccine (available from : http://www.merck.com/product/usa/pi_circulars/g/gardasil/gardasil_pi.pdf, accessed 2020 April 2), the systemic and serious adverse effects, namely the rate of autoimmune disorders, are evaluated comparing the group receiving Gardasil with only one group, receiving aluminium or placebo. By this way, any potential reactogenic effect of aluminium salts was masked.  Certainly, vaccine safety is assessed also in the post-marketing phase by means of pharmacovigilance systems, which can provide important indications on the incidence of AEFI in vaccinated subjects, which can be compared with unvaccinated subjects. However, this type of comparison is largely subject to various types of selection bias and to the diversity of non-randomized groups. The problem becomes even more serious in the case of adverse reactions with low incidence. For these methodological reasons, the application of the evidence from medical literature to assess causality should be used with great caution and should not become a cut-off argument to establish or exclude causality.”

 I hope thar after this requested implementation, the second version of the manuscript is considered as acceptable.

# Peer review: Reviewer response for version 1

**DOI:** 10.5256/f1000research.24947.r61073

**Published:** 2020-03-23

**Authors:** Jacob Puliyel

**Affiliations:** 1 Holy Family Hospital, New Delhi, Delhi, India

Paola Bellavite (Verona University, Italy) has reviewed the WHO’s causality classification of adverse events following immunization (AEFI) in an opinion piece. Using 3 case studies of AEFI deaths reported to the Italian Medicines Agency (Agenzia Italiana del Farmaco AIFA) he illustrates how application of the WHO algorithm is difficult and prone to error. 

 The glossary of the WHO manual (page vii) defines ‘causal association’ as a cause and effect relationship between causative factor and a disease with no other factor intervening in the process. Bellavite, quite rightly, feels that this is a wrong approach. 

 He lists (Table 1), a series of genetic disorders that have been associated with tendency to develop AEFI. Using the WHO definition, a causal association with vaccine would be denied because of the genetic factor intervening in the process. Bellavite has proposed that a ‘consistent association of the adverse event with the vaccine’ must only be excluded when the presumed ‘other cause’ independently (without interaction with the vaccine) causes the AEFI. This makes good sense.

 At step 3 along the mandatory path of the algorithm, the question is: “Is there strong evidence against a causal association?” Bellavite correctly points out the impossibility of proving a negative. “Lack of evidence of association” may mistakenly be considered as “evidence of lack of association” or evidence against a causal association. 

 It is interesting that the WHO manual quotes an Institute of Medicine (IOM) report on a study looking at a possible relationship between SIDS and vaccines and which concluded that vaccines did not cause SIDS. Will this loose use of the generic term ‘vaccines’ mean that, hereinafter, no vaccine can have a causal association with SIDS or does this statement relate only to the vaccines examined by the IOM. The problem with proving a universal negative is that a single instance of a positive association can negate all the previous experiences and studies. Such a universal negative assertion is seldom made in scientific literature.

 These are important issues that have been raised. 

 Till 2013 the WHO used the Brighton classification of AEFI (1) and causal association was classified as ‘certain’: ‘probable’: ‘possible’: ‘unlikely’ and ‘unclassifiable’. The categories were revised in 2013 (2). F1000research published a critique of this classification by the reviewer (3). The Second Edition of Revised AEFI classification was published (with minor changes) in 2018 (4).

 In a communication in the British Medical Journal, Chandler of the Brighton Collaboration has asserted (5) and I quote extensively (italicized):

*“The WHO AEFI causality assessment was developed by the Vaccines Safety Group at the WHO with the support of the Global Advisory Committee on Vaccine Safety. The target user group for this classification system are persons working in countries in whom vaccines are administered via WHO sponsored public health programmes. Those persons are largely concerned with the detection of "signals" of changes in frequency of the more common, expected events which could suggest vaccine quality-related problems, immunisation errors, or multi-use vial contamination, etc. At the current time, most AEFI reports collected and assessed with the WHO AEFI Causality Classification remain within the databases of the public health programmes and are not forwarded into the databases of the national pharmacovigilance centres of most lower and middle income countries.*

*In contrast, more general guidance for causality assessment, such as the WHO-UMC causality criteria and the Naranjo algorithm, were developed by various groups working within the greater field of pharmacovigilance. The target user groups for these classification systems are those persons working within national pharmacovigilance centres, usually working within or collaboratively with national regulatory centres, and responsible for post-marketing safety surveillance of both drugs and vaccines used within their countries. Within such centres adverse event reports for drugs and vaccines are often maintained within a single database (one notable exception being the USA), and causality assessment is approached in a similar way for all products. Detection of "signals" within the database can be conducted qualitatively (on a "case-by-case" basis) and/or quantitatively (via statistical screening ). Higher income countries which do not rely upon implementation of vaccine administration through WHO public health programmes will handle reports of AEFI through these national pharmacovigilance centres.*

*Furthermore, it is worth noting that most reports of AEFI contained with Vigibase, the database of individual case safety reports for the WHO Programme of International Drug Monitoring, are from countries who channel reports of AEFI through their national pharmacovigilance system, and therefore most reports within the global database have not been subject to WHO AEFI causality assessment.*

*Taking the specific example of narcolepsy, reports of this condition in association with Pandemrix, an H1N1 pandemic vaccine, were initially received into the national pharmacovigilance centres of Sweden and Finland, and therefore they were not subject to causality assessment by the WHO AEFI classification system. This signal was detected, in fact, because these clusters of reports in young children were "unexpected" , by both the reporting physicians (based upon their clinical practice) and by the regulators (based upon the expected reporting patterns within their national databases of suspected adverse drug reactions).*

*The current system referred to as "robust" within this analysis therefore refers to practice of vaccine pharmacovigilance by national pharmacovigilance/regulatory centres, not that of national immunisation centres routinely utilising the WHO-AEFI causality classification system.”*

 It seems from this that the WHO causality assessment is meant for poor and developing countries and most reports within the global database for pharmacovigilance have not been subject to WHO AEFI causality assessment. it is interesting that the cases cited by Bellavite, the AEFI deaths reported to the Italian Medicines Agency were subjected to the WHO AEFI assessment.

 The point that Bellavite makes is that compensation may be denied to families who die after vaccination, utilizing this classification. It will be intriguing to know if this classification is used in Italy  to deny compensation but, as a ‘developed country’, it uses a second system  for pharmacovigilance. 

 The 2018 revised manual says it was ‘scientifically evaluated’ looking for inter-rater reliability between teams from India and Zimbabwe. It was not examined against any gold standard. If two populations consistently perceive the world is flat, it does little validate the ‘scientific’ reliability of that perception. 

 The paper by Bellavite is an important addition to the literature. However, it can be improved by extensive revision. The language can be improved and corrected in many places. This reviewer has often had to resort to such help, for his scientific communications.

 1. Introduction: The author writes that AEFI harms a few “unlucky” individuals. The term related to luck put within quotation marks is best deleted in a scientific communication.

 2. Page 4 Innate immune response. It is not clear what the author wants to convey about the risk of fever after MMR. He says this is more in children under 35 months compared to children older than 4 years of age.

 I am not able to understand what this has to do with the AEFI classification and why this is brought up here. 

 3. Page 5 The author suggests that some autoimmune disorders may be associated with immunization but it is not specified what changes in the AEFI classification will help to identify the role that vaccines play. 

 4. The list of genetic disorders listed in Table 1 is useful as a ready-reckoner, but for that, it must be as exhaustive as possible. I am not an expert in this area but the association of AEFI with mitochondrial disorders is one that I recognize is missing from the list (Poling PMID 16566887) 

 5. Page 7 The text says “It is important to point out vaccines may safely be administered in children with Di George syndrome.” 

 Why is it important to state this? There are a whole host of genetic disorders where vaccines can be administered with impunity. Why has Di Gorge been singled out to be declared as safe

 6. Micrbiome - The relevance of the paragraph on the microbiome is also not clear in the context of AEFI classification.

   

 7. Page 8 The first two paragraphs: It is not clear what the author wants to convey and how it relates to the WHO AEFI classification method

 8. Page 9 Top paragraph not clear

 9. So also Note 3 The Literature (Delete ‘Note 3’ from the paragraph heading)

 The content of this paragraph is not clear

 10. Page 10 Second last paragraph

 The author writes 

 “The most obvious case of a possible overlap between autism spectrum symptoms and another disease, surely caused by vaccine adjuvants, is the macrophagic myofasciitis.“

 A little more elaboration would be helpful because macrophagic myofasciitis is a relatively new syndrome associated with vaccine aluminium adjutants and its association with cognitive disorders is known even less.

Is the topic of the opinion article discussed accurately in the context of the current literature?

Partly

Are arguments sufficiently supported by evidence from the published literature?

Partly

Are all factual statements correct and adequately supported by citations?

Partly

Are the conclusions drawn balanced and justified on the basis of the presented arguments?

Partly

Reviewer Expertise:

Pediatrics

I confirm that I have read this submission and believe that I have an appropriate level of expertise to confirm that it is of an acceptable scientific standard, however I have significant reservations, as outlined above.

## References

[1] : Adverse Events Following Immunization(AEFI): Causality Assessment.2005; Reference source

[2] : Causality assessment of an adverse event following immunization (AEFI).2018; Reference source 24461168

[3] : Revised World Health Organization (WHO)'s causality assessment of adverse events following immunization-a critique. *F1000Res*.2018;7: 10.12688/f1000research.13694.2 243 10.12688/f1000research.13694.2 30026925PMC6039921

[4] : Modernising vaccine surveillance systems to improve detection of rare or poorly defined adverse events. *BMJ*.2019; 10.1136/bmj.l2268 10.1136/bmj.l2268 31151960

# Response

**Published:** 2020-04-02

**Authors:** Paolo Bellavite

**Affiliations:** University of Verona School of Medicine, Italy

I thank Dr. Puliyel for his reviewer’s report that allows me to improve the paper and specify some aspects better. Here I respond to his observations point-by-point and highlight the new parts inserted in the text

 I appreciate that the most important proposal of the paper has been accepted. Rightly dr. Pulijel recalls that the same F1000research Journal published a critique of this classification, which I appropriately cited (quotation 158 of the first version). There are several points raised by the Reviewer that I have addressed in the revised paper:

 1.            Dr. Puliyel has cited a long and interesting declaration of dr. Chandler including the sentence “The target user group for this classification system are persons working in countries in whom vaccines are administered via WHO sponsored public health programmes “ and suggested that “ It seems from this that the WHO causality assessment is meant for poor and developing countries and most reports within the global database for pharmacovigilance have not been subject to WHO AEFI causality assessment. it is interesting that the cases cited by Bellavite, the AEFI deaths reported to the Italian Medicines Agency were subjected to the WHO AEFI assessment.”

 Response: This is a straightforward point that was raised also by dr. Chandler as Reviewer and I have modified the text according your and her suggestions. I have given only one response,  under her comments to the paper. Moreover, connected with Chandler’s comments, I have added to the Discussion a paragraph to better clarify the limitations of WHO algorithm for evaluating the responsibility of vaccines in multifactorial AEFI: “The WHO causality assessment is mainly constructed on a concept of direct ‘a cause-and-effect relationship”, thus dismissing the multifactorial nature of inflammatory and immune phenomena. By underestimating interacting causes, the  method classifies a possible association as “inconsistent” when there is another cause and as “indeterminate” when the role of the vaccine can’t be excluded, but there is no proof that it is “the” cause. Using this definition of causal association, many adverse events, where the vaccine plays a role as con-cause, remain unrecognized. Others (Puliyel and Naik, 2018) have noted that, according to this scheme, an acute cardiac decompensation after influenza vaccination in an elderly person with chronic cardiac failure might not be considered as causally related to the vaccine. Similarly, sudden death after vaccination of an infant with pre-existing heart disease might not have relationship with the vaccine. Furthermore, the contribution of vaccine in precipitating encephalopathy in patients who are susceptible on account of genetic factors will also not be considered. If this type of problem occurs, in addition to causing detriment to a injured person, it leads to an overall underestimation of the risks of a given vaccine.

 2.            The point that Bellavite makes is that compensation may be denied to families who die after vaccination, utilizing this classification. It will be intriguing to know if this classification is used in Italy  to deny compensation but, as a ‘developed country’, it uses a second system  for pharmacovigilance.

 Response: In Italy, the WHO classification is considered the “gold standard” in the evaluation of AEFI originating from pharmacovigilance reports, so it is normally the only one that is used for causality assessment. Of course, if people who believe they have suffered unrecognized damage from vaccination appeal to a court of justice, a much more detailed assessment follows, where the expert consultants of the parties are challenged with all the available documentation. I have added this further explanation to the Discussion in the paragraphs concerning the compensation.

 3.            The 2018 revised manual says it was ‘scientifically evaluated’ looking for inter-rater reliability between teams from India and Zimbabwe. It was not examined against any gold standard. If two populations consistently perceive the world is flat, it does little validate the ‘scientific’ reliability of that perception.

 Response: I agree that this is one of the several problems raised by the manual and I hope that my paper will contribute to highlight that considering the WHO classification as an universal “gold standard” would be an error.

 4.            Introduction: The author writes that AEFI harms a few “unlucky” individuals. The term related to luck put within quotation marks is best deleted in a scientific communication

 Response: OK I agree and have removed this word

 5.            Page 4 Innate immune response. It is not clear what the author wants to convey about the risk of fever after MMR. He says this is more in children under 35 months compared to children older than 4 years of age.  I am not able to understand what this has to do with the AEFI classification and why this is brought up here.

 Response: I wanted to report in one sentence the quantitative value of the risk of febrile seizures according to the literature and in the following one the risk of MMRV compared to MMR alone. I believe it is important to offer readers an estimate of what the frequency of one of the most serious adverse reactions is, because the evaluation of causality must also take into account, among the other factors, also the probability that a certain AEFI can occur in a specific age group. 

 6.            Page 5 The author suggests that some autoimmune disorders may be associated with immunization but it is not specified what changes in the AEFI classification will help to identify the role that vaccines play.

 Response: This topic is very important but does not have an easy solution. In fact, as has been reported in the text, autoimmune diseases and in general chronic post-vaccination chronic syndromes are due to multiple factors intertwined with each other and, moreover, they can arise weeks or months after vaccination. However, to answer the reviewer’s correct question, I have added a paragraph on autoimmune diseases to the "final categorization" in Discussion (Note 4.) and on page 5 I have added a sentence that refers to the final discussion: “The implications of these concepts in the causality assessment are discussed in a later chapter (see Note 4.)” The added paragraph is the following: “As mentioned, multifactorial diseases, such as autoimmune diseases, are often conditioned by various genetic and acquired factors. In these cases, the role of vaccination could be to slatentize a predisposing condition, which would have led to the disease slower or would not even appear. If this is the case, it is probable that the case study will neither confirm nor deny the role of the vaccine, so that the causality assessment would come to the conclusion of an "undetermined" association. Obviously, this procedure, if applied systematically to a series of cases, would lead to an underestimation of the etiological role of vaccines in autoimmune diseases. To overcome this vicious circle, in the final categorization (phase IV), the probability that the vaccine played a role in determining the event could be assessed and scored, taking into account the other possible factors involved. In this way, it would prevent information on the partial role of the vaccine, obtained from a particular case, from being lost in the study of a series of cases.”

 7.            The list of genetic disorders listed in Table 1 is useful as a ready-reckoner, but for that, it must be as exhaustive as possible. I am not an expert in this area but the association of AEFI with mitochondrial disorders is one that I recognize is missing from the list (Poling et al., 2006)

 Response: I thank of this suggestion, I have added the indicated reference to Table 1. To the best of my knowledge, there are not further genetic traits which have been associated with AEFI.

 8.            Page 7 The text says “It is important to point out vaccines may safely be administered in children with Di George syndrome.”  Why is it important to state this? There are a whole host of genetic disorders where vaccines can be administered with impunity. Why has Di Gorge been singled out to be declared as safe

 Response. Also this question is correct. The reason is that this is a genetic disorder of immune system (immunodeficiency), it is a notable exception of the rule that live vaccinas should not be administered to these children. In any case, to better clarify this point, in the revised version I have changed “in this context” (too generic) to “in the context of immunodeficiency diseases”

 9.            Microbiome - The relevance of the paragraph on the microbiome is also not clear in the context of AEFI classification.

 Response: Indeed, this point needs to be clarified. Thanks to the reviewer's question, I added this explanation at the end of the microbiome paragraph: “The role of microbiome is important from the perspective of susceptibility factors of AEFI, because it is possible that an alteration of the gut health, especially with the leak of endotoxins in the general circulation, increases the susceptibility to a stronger and more serious reaction to the immune stimulus represented by the vaccine . Under these predisposing conditions the plausibility that a serious inflammatory reaction may be triggered by a vaccination increases.”

 10.             Page 8 The first two paragraphs: It is not clear what the author wants to convey and how it relates to the WHO AEFI classification method

 R: This topic is central and has to do precisely with the complex nature of many reactions to vaccines. Also thanks to the review to the first version by Dr. David Legge, who appreciated the setting of the entire work based on the description of the typical features of complex systems, I believe that this difficult topic has been well developed. Since it is possible that the meaning of this point may still be a little obscure, I added an explanatory paragraph, which is more closely related to the AEFI causal assessment: “Therefore, in the case of diseases involving several possible causes and/or mechanisms, the purpose of AEFI classification cannot be to identify "the" determining cause, but it may be more correct to try to establish with what probability one or more the factors involved (vaccines, genetic or epigenetic traits, previous or concomitant infections, drugs, age, gender, nutrition and metabolism, etc.) may have contributed to the occurrence of the event. “

 11.          8. Page 9 Top paragraph not clear

 Response. I have added a further paragraph to better clarify the problem of time window: “The WHO guidelines consider this problem under the question "In this patient, did the event occur within a plausible time window after vaccine administration?" and in a note cite as a "detailed document" a book of the Institute of Medicine(Institute of Medicine, 2012). However, in that document there are no indications on the suitable time windows of autoimmune diseases or in general of chronic diseases following vaccinations. In some cases, causality is excluded by using rather short time windows. For example, the case of a man with symptoms of chronic inflammatory disseminated polyneuropathy that occurred 8 weeks after a tetanus toxoid vaccine(Hughes et al., 1996) is presented and it is argued that this interval is "too long". However, autoimmune diseases and chronic post-vaccination syndromes in general can occur several weeks or months after vaccination. In cases of fibromyalgia and chronic fatigue disease following hepatitis B vaccination (Agmon-Levin et al., 2014), the time interval between vaccination and the onset of symptoms was 38.6 days, but with a large time interval (+/- 79.4 days). In a systematic prospective case-referent study conducted to assess the risks of autoimmunity associated with HPV vaccines, a reasonable time window of 24 months for multiple sclerosis, connective tissue disease, type-1 diabetes, and thyroiditis was adopted (Grimaldi-Bensouda et al., 2017). This paper excluded an association between HPV vaccination and these disease, but an increased percentage of cases had personal or family (in first-degree relatives) history of autoimmunity (14.7% of cases versus 7.2 % of referent group, p <0.05), endorsing the importance of genetic susceptibility to vaccine adverse effects. Given the complexity and multifactoriality of chronic autoimmune diseases and the lack of precise references on the time frame of appearance of these diseases after vaccination, the possibility could be considered that, in an upcoming edition of the guidelines, it is specified that the time window for autoimmune diseases should be sufficiently large (e.g. 24 months(Grimaldi-Bensouda et al., 2017) ) to not exclude slow-onset cases, or that a restricted time frame should be applied only to AEFI with acute onset (for example: hyperthermia, febrile seizures, anaphylaxis).

 12.          So also Note 3 The Literature (Delete ‘Note 3’ from the paragraph heading) The content of this paragraph is not clear

 Response:  To clarify the meaning of the paragraph, at the beginning  I have added a sentence: ” In practice, if there is published evidence in literature that rejects a statistically significant association between a disease and previous vaccinations, this argument could be used to exclude in each particular case that vaccination may have caused the reported disease. The evidence at population scale is used at individual level.” I cannot delete the words "Note 3" because the heading helps to clarify that this paragraph connects with the third step of the algorithm and the whole chapter is divided into four notes in their order. 

 13.          Page 10 Second last paragraph. The author writes  “The most obvious case of a possible overlap between autism spectrum symptoms and another disease, surely caused by vaccine adjuvants, is the macrophagic myofasciitis.“ A little more elaboration would be helpful because macrophagic myofasciitis is a relatively new syndrome associated with vaccine aluminium adjuvants and its association with cognitive disorders is known even less.

 Response: This topic was developed in a previous chapter, where the pathogenic effects of aluminum on the central nervous system were described, including cognitive dysfunction, sensory disturbances, and motor retardation. I added this sentence to the discussion at that point mentioned by the Reviewer.

 I believe these changes help make the text clearer and I thank the Reviewer for that

 Reference List

 Agmon-Levin N, Zafrir Y, Kivity S, Balofsky A, Amital H, Shoenfeld Y. Chronic fatigue syndrome and fibromyalgia following immunization with the hepatitis B vaccine: another angle of the 'autoimmune (auto-inflammatory) syndrome induced by adjuvants' (ASIA). Immunol Res 2014; 60(2-3): 376-383.

 Grimaldi-Bensouda L, Rossignol M, Kone-Paut I, Krivitzky A, Lebrun-Frenay C, Clet J, Brassat D, Papeix C, Nicolino M, Benhamou P Y, Fain O, Costedoat-Chalumeau N, Courcoux M F, Viallard J F, Godeau B, Papo T, Vermersch P, Bourgault-Villada I, Breart G, Abenhaim L. Risk of autoimmune diseases and human papilloma virus (HPV) vaccines: Six years of case-referent surveillance. J Autoimmun 2017; 79: 84-90.

 Hughes R A, Choudhary P P, Osborn M, Rees J H, Sanders E A. Immunization and risk of relapse of Guillain-Barre syndrome or chronic inflammatory demyelinating polyradiculoneuropathy. Muscle Nerve 1996; 19(9): 1230-1231.

 Institute of Medicine. Adverse effects of vaccines: Evidence and causality. The National Academies Press, Washington, DC 2012.

 Poling J S, Frye R E, Shoffner J, Zimmerman A W. Developmental regression and mitochondrial dysfunction in a child with autism. J Child Neurol 2006; 21(2): 170-172.

 Puliyel J, Naik P. Revised World Health Organization (WHO)'s causality assessment of adverse events following immunization-a critique. F1000Res 2018; 7: 243.

# Peer review: Reviewer response for version 1

**DOI:** 10.5256/f1000research.24947.r61078

**Published:** 2020-03-18

**Authors:** David Legge

**Affiliations:** 1 School of Public Health, La Trobe University, Melbourne, Vic, Australia

The author suggests that the 2018 WHO algorithm for causality assessment in cases of adverse events following immunisation (AEFI) may be overly mechanistic and may not do justice to the complexity of biological systems and the possibility of multifactorial causation.  

 He sets the scene regarding bio-complexity with reference to complex disease causation, the complexity of the immune response (including the genesis of autoimmunity), the role of genetic variants in modulating immune reactions, and the role of the microbiome.  

 He then examines the 2018 WHO algorithm considering four steps in some detail.  

- The first step in WHO's causal algorithm asks if there is 'strong' evidence for an alternative cause of the event. The author suggests that this either-or question precludes consideration of the possibility of an interaction between the alternative cause and the immunisation event. 
- The second question raised concerns the significance of temporal association (between immunisation and adverse event) in determining causality. The author argues, drawing particularly on immunopathology, that some pathways of causation could operate over weeks and months rather than hours and days.
- The third element of the algorithm which the author questions concerns the exclusion (with respect to causality) of cases where there is 'strong' evidence *against* a causal relation. The concern raised here concerns the meaning of 'strong' evidence in relation to the methodological limitations of such research. 
- The fourth element of the algorithm in question concerns the final classification of 'consistent with' or 'not consistent with' causality. Here the author returns to his concerns about the either-or logic upon which the algorithm rests and the need to properly accommodate multifactorial causation. 

In concluding his review the author proposes further development of the WHO algorithm to better accommodate multifactorial causation. He also comments on the links between AEFI causality assessment and access to compensation for people who have experienced harm after immunisation. He suggests that the appropriate standards for causal inference for compensation purposes might not be same as those for regulatory purposes. Finally the author returns to multifactorial causation, highlighting in particular, genetic influences and the need for further research including regulatory monitoring of genetic factors (and perhaps microbiomic factors). He suggests that closer attention to the interplay of these (potentially) contributory factors could contribute to more evidence-informed decision-making regarding exemptions from mandated (or highly recommended) immunisations. 

 This is a very thoughtful and well informed contribution to the continuing discussion of causal assessment in cases of AEFI. The author has presented a 'strong' case for closer attention to complexity and multifactorial causation in causality assessment of AEFIs. The distinctions he makes between the different contexts and purposes of causal assessment (regulation, compensation and exemption) are useful.

Is the topic of the opinion article discussed accurately in the context of the current literature?

Yes

Are arguments sufficiently supported by evidence from the published literature?

Yes

Are all factual statements correct and adequately supported by citations?

Yes

Are the conclusions drawn balanced and justified on the basis of the presented arguments?

Yes

Reviewer Expertise:

I am a generalist public health physician with a particular interest in global health policy. I have a long standing interest in complexity theory as applied to biological and social systems. However, I have approached this review with a policy perspective.

I confirm that I have read this submission and believe that I have an appropriate level of expertise to confirm that it is of an acceptable scientific standard.

# Response

**Published:** 2020-03-30

**Authors:** Paolo Bellavite

**Affiliations:** University of Verona School of Medicine, Italy

I warmly thank Doctor Legge for this positive comment on my work, of which he demonstrates that he has understood the fundamental meaning in the context of the complex vision of multifactorial diseases. His contribution is invaluable in preparing the second version and I will certainly take it into account

 Paolo Bellavite (Author)
